# Surveillance of bloodstream infections in pediatric cancer centers – what have we learned and how do we move on?

**DOI:** 10.3205/dgkh000271

**Published:** 2016-05-12

**Authors:** Arne Simon, Rhoikos Furtwängler, Norbert Graf, Hans Jürgen Laws, Sebastian Voigt, Brar Piening, Christine Geffers, Philipp Agyeman, Roland A. Ammann

**Affiliations:** 1 Pädiatrische Onkologie und Hämatologie, Universitätsklinikum des Saarlandes, Homburg, Germany; 2 Klinik für Pädiatrische Onkologie, Hämatologie und Immunologie, Universitätskinderklinik, Heinrich-Heine-Universität, Düsseldorf, Germany; 3 Klinik für Pädiatrie m. S. Onkologie / Hämatologie / Stammzelltransplantation, Charité – Universitätsmedizin Berlin, Germany; 4 Institut für Hygiene und Umweltmedizin, Charité – Universitätsmedizin Berlin, Germany; 5 Pädiatrische Infektiologie und Pädiatrische Hämatologie-Onkologie, Universitätsklinik für Kinderheilkunde, Inselspital, Bern, Switzerland

**Keywords:** pediatric oncology, bloodstream infection, Broviac, Port, surveillance

## Abstract

Pediatric patients receiving conventional chemotherapy for malignant disease face an increased risk of bloodstream infection (BSI). Since BSI may represent an acute life-threatening event in patients with profound immunosuppression, and show further negative impact on quality of life and anticancer treatment, the prevention of BSI is of paramount importance to improve and guarantee patients’ safety during intensive treatment. The great majority of all pediatric cancer patients (about 85%) have a long-term central venous access catheter in use (type Broviac or Port; CVAD). Referring to the current surveillance definitions a significant proportion of all BSI in pediatric patients with febrile neutropenia is categorized as CVAD-*associated* BSI. This state of the art review summarizes the epidemiology and the distinct pathogen profile of BSI in pediatric cancer patients from the perspective of infection surveillance. Problems in executing the current surveillance definition in this patient population are discussed and a new concept for the surveillance of BSI in pediatric cancer patients is outlined.

## Zusammenfassung

Pädiatrisch-onkologische Patienten unter einer konventionellen Chemotherapie sind einem erhöhten Risiko für Blutstrominfektionen (BSI) ausgesetzt. Da diese mit einer erheblichen Gefährdung und Belastung der Patienten einhergehen, ist die Prävention von BSI ein wichtiger Bestandteil des Gesamtkonzepts zur Gewährleistung der Patientensicherheit während der intensiven Behandlung. Die Mehrzahl der Patienten (ca. 85%) hat einen langfristig implantierten zentralen Venenkatheter vom Typ Broviac oder Port (CVAD); nach den gängigen Definitionen der Infektionssurveillance sind daher ein erheblicher Anteil der BSI bei Patienten mit Fieber und Granuolozytopenie „Gefäßkatheter-assoziiert“ (CABSI). In dieser Übersicht zum Stand des Wissens werden die Epidemiologie und das besondere Erregerprofil von BSI bei pädiatrisch onkologischen Patienten aus der Perspektive der Infektionssurveillance beschrieben. Probleme bei der Anwendung der gängigen Definitionen werden aufgezeigt und es wird ein Vorschlag für ein neu aufgelegtes Erfassungsmodul für die Surveillance von BSI bei kinderonkologischen Patienten vorgestellt.

## List of abbreviations

- ALL – acute lymphoblastic leukemia
- AML – acute myeloblastic leukemia
- ARDS – acute respiratory distress syndrome
- BSI – bloodstream infection
- CA-BSI – vascular catheter-associated bloodstream infection
- CDC – Centers for Disease Control and Prevention
- CFU – colony forming units
- CoNS – coagulase-negative staphylococci
- CR-BSI – bloodstream infection with the vascular catheter as the most probable source
- CVAD – long-term tunneled or subcutaneously implanted central venous catheter, type Port, Broviac or Hickman
- FCH – fluoroquinolones
- GPOH – German Society of Pediatric Oncology and Hematology
- GVHD – graft-versus-host disease
- MBI-LCBSI – mucosal barrier injury-associated laboratory-confirmed BSI
- MDS – myelodysplastic syndrome
- MNS – face mask (surgical grade)
- MRGN – multidrug-resistant Gram-negative pathogen
- MRSA – methicillin-resistant *Staphylococcus aureus*
- NFC – needleless (needle free) connecting device
- NI – nosocomial infection
- NICU – neonatal intensive care unit
- PICU – pediatric intensive care unit
- PJP – *Pneumocystis jrovecii* pneumonia
- POC – pediatric oncology treatment center
- SCT – stem cell transplantation
- VGS – viridans (alpha-hemolytic) streptococci
- VRE – vancomycin-resistant enterococci (in most cases: *E. faecium*)
- 2 MRGN NeoPäd – Gram-negative pathogen, in vitro resistant to piperacillin and extended spectrum cephalosporins (cefotaxime, ceftriaxone, ceftazidime)
- 3 MRGN – Gram-negative pathogen, in vitro resistant to 3 of 4 antibiotic classes utilized for empirical treatment of systemic infection in adult high risk patients (piperacillin, extended-spectrum cephalosporins, carbapenems and fluoroquinolones)
- 4 MRGN – Gram-negative pathogen, in vitro resistant to 4 antibiotic classes utilized for empirical treatment of systemic infection in adult high risk patients (piperacillin, extended-spectrum cephalosporins, carbapenems and fluoroquinolones)

## Background

In children with malignancy receiving conventional anticancer chemotherapy, bloodstream infections (BSI) caused by bacterial pathogens represent nearly half of all nosocomial infections (NI) in which a pathogen can be isolated [1], [2], [3], [4], [5], [6], [7], [8], [9], [10], [11], [12]. In contrast to other high-risk pediatric patient populations [13], [14], [15], [16], [17], the great majority of all pediatric cancer patients (about 85%) have a long-term central venous access catheter in use (type Broviac, Hickman or Port; CVAD) [18], [19], which is surgically implanted early during induction treatment [20]. Due to this high utilization rate of CVADs, a significant proportion of all BSI in this population is *associated* with a CVAD. Table 1 (Tab. 1) shows a number of prospective studies in which different protocols of prospective surveillance for BSI have been followed in pediatric cancer patients. In most malignancies deriving from or with extensive involvement of the bone marrow, the intensive chemotherapy and – in some patients – radiotherapy of the underlying disease result in a severely reduced number of granulocytes in peripheral blood cell counts (neutropenia; number of granulocytes in peripheral blood <0.5x10^9^/L or <1.0x10^9^/L and no differential count available). Neutropenia negatively affects the capacity of the child to defend against bacterial and fungal pathogens, and fosters the risk of BSI [21]. On the other hand, BSI have been observed in pediatric cancer patients without neutropenia at the onset of symptoms (e.g. fever) [11], [22], [1], [6]. This is the case in nearly half of all pediatric cancer patients with bacteremia (detection of a pathogen in blood cultures in a symptomatic child) who do not show clinical signs of sepsis [1]. 

Beyond the acute phase after stem-cell transplantation [23], [24], the surveillance of BSI in pediatric cancer patients should not only be performed in those patients with neutropenia. It seems more reasonable to adjust the events (BSI) not to days of neutropenia, but to 1000 in-patient days or to 1000 utilization days for CVADs. 

Notwithstanding, the clinical severity of the BSI [25] and the risk of severe and even life-threatening complications [1], [26] are significantly influenced by the severity and duration of neutropenia after the onset of the infection.

### Mucositis

Intensive chemotherapy (in particular high-dose methotrexate, anthracyclines, high-dose cytarabine, high-dose etoposide) and radiotherapy may cause injury to mucosal surfaces in the mouth, oropharynx and deeper parts of the gastrointestinal system (mucosal barrier injury; mucositis) [27], [28]. Both neutropenia and mucosal barrier injury increase the risk of translocation from the mucosal surface into the bloodstream [29]. This concerns most prominently viridans group streptococci (VGS), Enterobacteriaceae (*E. coli*, *Klebsiella* spp., *Enterobacter* spp.), and enterococci (*E. faecium* and *E. faecalis*) [30]. In the case of substantial failures in terms of prevention (hand hygiene, disinfection of IV connections or three-way stopcocks before any access) [18], infection with those pathogens may be exogenous in origin; the pathogens contact the inner surface of the CVAD via healthcare workers’ hands or through contaminated substances and infusions [31], [32]. The latter may be even more probable in patients with severe mucositis, who are in need of many supportive-care manipulations of their CVAD (e.g. parenteral nutrition, continuous analgesic infusion).

### Antimicrobial prophylaxis

Most pediatric oncology patients receive antimicrobial prophylaxis (cotrimoxazole at least once a week) to prevent *Pneumocystis jirovecii* pneumonia [33], [34], [35], [36], [37], [38]. In contrast to adults with leukemia and a high risk of bacterial translocation from the gut [39], antimicrobial prophylaxis with fluoroquinolones (FCH) is only rarely used in children and adolescents with cancer [40], [41]. In contrast, oral treatment with FCH (e.g., as sequential oral treatment after 48–72 hours of IV treatment, or after stem-cell transplantation) has recently been chosen more often as an alternative to other oral antibiotics even in pediatric cancer patients [42], [43]; however, we are not aware of any data describing the use of FCH in detail in this population in German pediatric oncology centers (POC). The historical selective oral decontamination concept with non-absorbable antibiotics, such as colistin, oral gentamicin or paromomycin [44], is no longer used by most German POCs due to a lack of scientific evidence for efficacy and compliance problems [45]. 

In patients with acute myeloblastic leukemia, some POCs administer penicillin as prophylaxis against VGS infection between chemotherapy cycles [46]. In addition, some POCs use teicoplanin infusions 3 times per week [47], [48]. Since there is still no consensus, both preventive strategies have been outlined in the German AML BFM Protocol (Version 01.04.2012 Chapter 8.2.5 p.41).

Felsenstein et al. performed a retrospective case series analysis to investigate the advantages and disadvantages of prophylactic FCH (ciprofloxacin) in pediatric patients with AML (n=45, 2008–2012; Children’s Hospital Los Angeles) [49]. The analysis revealed a probable benefit in terms of less BSI caused by Gram-negative pathogens (13.4% vs. 4.7%); on the other hand, the incidence of BSI due to Gram-positive pathogens increased significantly (28% vs. 14%). The use of ciprofloxacin prophylaxis increased the risk of BSI due to VGS. Eventually, the incidence of BSI was equal in both retrospectively compared groups (35.9% vs. 31.5%). No influence on mortality was detected.

### Spectrum of bacterial pathogens of BSI in pediatric cancer patients

The spectrum of pathogens derived from blood cultures in symptomatic pediatric oncology patients shows some differences compared to BSI in other pediatric populations. Coagulase-negative staphylococci (CoNS) account for 20% to 30% of all Gram-positive pathogens. It is probable that a significant proportion of these opportunistic pathogens obtain access to the bloodstream through the CVAD and its maintenance care.

Since more than half of these CoNS display in-vitro resistance to methicillin, these BSI foster the extensive use of glycopeptides in POCs [50], [51]. Depending on the subgroup of patients, between 15% [6] and 26% [3] of all BSI are caused by viridans group streptococci; (VGS) [52]. In this regard, VGS are more prevalent than *S. aureus* (9%) as pathogens detected in BSIs [6]. In contrast, VGS represent less than 2% of all pathogens detected in blood cultures of pediatric intensive-care patients [14]. In a significant proportion of all cases (up to 15%; in particular *S. mitis*, often penicillin resistant) [53] BSI caused by VGS is accompanied by clinical sepsis and pneumonia or acute respiratory failure (ARDS) [54], [55]. BSI caused by VGS have significantly more often been reported in patients with AML, induction chemotherapy with cytarabine, neutropenia and mucositis [56], [57], [58]. Pneumococci, which represent the most common pathogens in community acquired sepsis in pediatric patients without underlying malignancy [59], [60], are detected in only 2% of all BSI in POCs [6]. Pediatric patients with ALL seem to face an increased risk *S. pneumoniae* BSI during maintenance treatment, since immunization against invasive isolates is regularly refreshed 4 months after the end of chemotherapy [61]. The most common Gram-negative pathogen detected in blood cultures of symptomatic pediatric cancer patients is *E. coli* [6], [1], [3], followed by other Enterobacteriaceae (e.g. *Enterobacter* spp., *Klebsiella* spp.) and non-fermenters such as *P. aeruginosa* (6–7% in most studies) [6], [44].

### Pathogens with in vitro resistance against commonly used antibiotics

In recent studies of nosocomial BSI performed in POCS in Germany, Switzerland and the Netherlands, the proportion of bacterial pathogens which display in vitro multidrug-resistance to commonly used antibiotics in this setting (MRE) [62], [63] (MRSA, VRE, MRGN) has been consistently low [1], [3], [4], [6]. Simon et al. (2001–2005, 7 POCs in Germany and Switzerland) analyzed 138 BSI (145 isolates) and found no MRSA, 2 VRE (1.5% of all BSI) and only two cases with 2 MRGN NeoPäd *[Please refer to list of abbreviations.]* (1.5% of all BSI; *K. pneumoniae* and P. aeruginosa, resistant in vitro to piperacillin and extended spectrum cephalosporins) [1]. In this study, the attributable mortality of nosocomial infections in POCs was 3%; 6 patients died due to invasive aspergillosis and 2 because of clinical sepsis and multi-organ failure without any pathogen detected in blood cultures.

Miedema et al. (2004–2007; 2008–2011; Groningen, Amsterdam, Bern) did not find a single case of BSI due to MRSA or VRE among 248 Isolates (202 BSI) [3]. Some of the Gram-negative pathogens displayed multidrug resistance in vitro due to the production of an extended-spectrum beta lactamase (ESBL; this is comparable to the 2 MRGN NeoPäd definition); in addition, 3 *P. aeruginosa* isolates with in vitro resistance to imipenem/cilastatin were found. In no case was the detection of MRE related to a fatal outcome (attributable mortality 0.5%, n=1). In this study, the proportion of Gram-negative pathogens resistant to FCH was higher in patients exposed to ciprofloxacin [3/7 (43%) vs. 25/28 (89%), p=0.044]. Interestingly, FCH resistant Gram-negative pathogens were detected in POCs using ciprofloxacin as prophylaxis even in patients without direct exposure to FCHs in their medical history. Besides sources in the outpatient setting (e.g., pets, pet food, contaminated meat from industrial breeding of animals) [64], [65], the possibility of nosocomial transmission in a POC must be taken into account [41].

In a recently published surveillance study by Ammann et al. [6] (Switzerland, Germany), no MRSA was found in 179 BSI (185 isolates); the study group described a single BSI due to VRE (0.6% ) and 2 BSI due to 2 MRGN NeoPäd (1.1%; one *E. coli*, and one *E. cloacae*). Overall attributable mortality was 1.8% (3/179 BSI). One of those children, in whom the cause of death was related to the BSI, was suffering from a sepsis syndrome caused by an *E. cloacae* isolate which expressed an extended-spectrum beta lactamase. This child had been empirically treated with piperacillin-tazobactam and gentamicin. Unfortunately, the isolate was resistant against both first line antibiotics. 

Haeusler et al. retrospectively investigated 280 Gram-negative BSI in 210 pediatric cancer patients (Royal Children’s Hospital, Melbourne, 2003–2010) [66]. The most prevalent species detected in blood cultures were *E. coli*, *Klebsiella* spp., and *Enterobacter* spp. Out of 280 BSI, 42 (15%) were caused by MRGN. This study revealed independent risk factors for BSIs caused by MRGN: high-intensive chemotherapy (autologous SCT; OR 3.7, CI95 1.1–11.4), nosocomially acquired BSI (OR 4.3; CI95 2.0–9.6), and the presence of MRGN colonization or infection during the preceding 12 months (OR 9.9, CI95 3.8–25.5). Patients with BSI due to MRGN infection had a significantly prolonged length of stay in the hospital (plus 9.5 days) as well as in the PICU (plus 2.2 days), and were more often in need of mechanical ventilation (15% vs. 5.2%). Differences in mortality between the MRGN and the comparator group were not statistically significant.

From an external perspective, it may be too easy to reach the conclusion that BSIs due to MRE [62], [63] are not only extraordinarily rare but also have no significant negative impact on outcomes in POCs. This would be a grave misinterpretation, dangerous from the perspective of the individual patient. Some patients in POCs show a number of risk factors predisposing for colonization and infection with MRE [67], [68]; in our experience, patients transferred from high-prevalence countries should always be allocated to this group (e.g., from southern and eastern Europe, Syria, Arab countries, North Africa). In addition, a growing number of studies describe complicated and protracted clinical courses in pediatric cancer patients with fever and BSI, in whom the primary treatment was not adequate in terms of in vitro resistance of the responsible pathogen [69], [70], [66].

Recent reports and case series from POCs in Italy are extremely alarming. These POC face an increasing prevalence of 4 MRGN [63] *P. aeruginosa* [71], [72]. 

Caselli et al. retrospectively evaluated data from a multicenter survey in Italian POCs (2000–2008). This survey detected 127 pediatric cancer patients (in 12 POCs) with a BSI caused by *P. aeruginosa*. Of these, 31% were caused by 4 MRGN *P. aeruginosa*. Overall mortality was 19.6% (25/127), with 36% (14/39) mortality in the 4 MRGN cases vs. 13% (11/88) in those BSI without a multidrug-resistance pattern. In multivariate analysis, the 4 MRGN status of the isolates was a significant independent risk factor for a fatal outcome [72]. Cioffi Degli Atti et al. [71] reported an outbreak of carbapenemase-positive, phenotypical 3 or 4 MRGN *P. aeruginosa*, which were eventually detected in 27 patients. Twelve of 27 children experienced a BSI/sepsis, 6 experienced other focal infections, and 9 of 27 were only colonized with the outbreak strain (infection rate 67%). BSI most often developed during periods of neutropenia. Eight of 12 children with BSI died related to the infection (attributable mortality 67%). The local infection control strategy involved active surveillance cultures and additional isolation procedures. This resulted in a reduction of the incidence density (new detections) from 0.72 to 0.34/1000 in-patient days. The authors suggest introducing the screening of all pediatric cancer patients in Italy for colonization with 3 or 4 MRGN to the routine prevention efforts to reduce nosocomial transmission and infection.

Unfortunately, there is currently no feasible and effective decolonization regime available for pediatric cancer patients with gastrointestinal colonization with MRGN isolates. In this regard, patients remain colonized for the whole duration of their intensive treatment. This observation is strongly related to individual consequences (e.g., determining the best empirical antimicrobial treatment in case of fever with or without neutropenia [73]) and to precautions considering hospital hygiene and transmission control [68], [74]. In this field, many questions regarding the most feasible prevention strategy are still a matter of ongoing discussion.

### Negative impact of BSIs

Bacterial BSI may represent an acute life-threatening event in patients with profound immunosuppression [75]. Patients are immediately hospitalized and treated with i.v. antibiotics and supportive care measures for at least 72 hours [76], [77], [78].

This concept primarily focuses on patient safety, but may result in additional reduction of quality of life in pediatric cancer patients and their families [79], [80]. The direct cost of treatment is significantly increased in patients with BSI; a very conservative calculation from a German POC revealed that additional expenses of at least € 4,400 have to be allocated to each event [81], [82]. Empirical broad-spectrum antimicrobial treatment of pediatric cancer patients with fever and neutropenia fosters the selective pressure for MRE in POCs [83], [84], [85]. In individual patients, the risk of antibiotic-associated diarrhea and other *C. difficile*-associated diseases increases following antibiotic treatment of BSIs [86]. Ultimately, each BSI may result in a delay of chemotherapy and reduced dose intensity, with negative consequences for long-term remission of the underlying malignancy.

## Problems regarding the allocation of a BSI to the vascular catheter (CVAD)

### Two separate blood culture sets?

In most patients with a single lumen CVAD (Broviac or Port) who are suffering from fever and neutropenia, antibiotic treatment is started soon after one set [*One set refers to an aerobic and an anaerobic blood culture bottle.*] of blood cultures has been taken from the CVAD [75]; a second set of blood cultures is drawn 12 to 24 h later, in particular in patients with ongoing fever. Thus, the interpretation of common skin flora, such as CoNS, *Corynebacteria* spp. or *Propionibacteria* spp., growing in blood cultures drawn from a symptomatic pediatric cancer patient may be challenging. The CDC criterion of “growth of potential contaminants (e.g., CoNS) in at least two independently drawn blood cultures” [87] is often not fulfilled in pediatric cancer patients with BSI. If the CDC criteria for BSI are strictly followed, a significant proportion of all BSI in pediatric cancer patients will be lost to surveillance issues due to this definition alone.

It has been proven that the proportion of positive blood cultures increases as a result of taking initial blood samples not only from the CVAD but additionally from a peripheral vein; the magnitude of this higher yield was 12% [88] to 18% [89] in recent studies. 

In spite of these observations, the sampling of peripheral venous blood cultures is not recommended in the current guidelines of the German Society of Pediatric Oncology and Hematology [18], [90], due to inconvenience and anxiety on the patients’ part related to an additional peripheral venous puncture. As a consequence of the missing second independently drawn blood culture, in accordance with standard CDC definitions [87], Kelly et al. [10] did not include a significant proportion of all detected BSI in pediatric cancer patients in their analysis. Subsequently, CoNS were no longer the most prevalent pathogens, but ranked fourth place (7%) behind enterococci, *S. aureus* and VGS. In the study by Choi et al., the corresponding change in definition “reduced” the incidence of BSI by 18.6% [91]. 

Surveillance protocols for BSI in pediatric cancer patients should evaluate all positive blood culture sets together with the clinical assessment of the attending pediatric oncologists. In the multicenter Oncoped studies (Germany and Switzerland), BSIs due to CoNS were counted as real BSI if CoNS grew in both bottles of the initial blood culture set and the attending pediatric oncologists adjusted antimicrobial treatment to this result (clinical assessment) [6], [1]. The treatment team may eventually lose confidence in surveillance protocols which do not take the clinical assessment of the attending physicians into account.

### Is the CVAD the source of the BSI?

In all pediatric cancer patients with fever but without a clinical focus of the infection, the question remains as to whether the BSI originated from the CVAD. Since in most cases, initial blood cultures are only drawn from the CVAD, it is not possible to determine a *differential time to positivity* (DTP) [89]. Most BSI are treated successfully in situ with antibiotics administered via the CVAD [92], [76]. Therefore, the CVAD is removed only in a minority of all cases during the course of the infection, making the tip of the CVAD unavailable for microbiological examination (e.g., with the Maki method) [51], [93]. 

Any recommendation to draw additional peripheral venous blood cultures (in addition to at least one blood culture set taken from the CVAD) will probably not be followed by the majority of POCs in Germany, since currently 90% of all POCs only take blood cultures from the CVAD [19], [34]. This practice has been implemented for many years to reduce pain, inconvenience and anxiety in pediatric cancer patients with fever and neutropenia [94].

Before any sampling of blood from the CVAD, a thorough disinfection of the catheter hub is recommended, usually with an alcoholic disinfectant (short dwell time of 15–30 seconds) [95], [18], [96]. Given a double-lumen Broviac/Hickman, this procedure is performed for both lumina (one blood culture set from each lumen) [97], [98]. Referring to a survey from 2013 [19], only 7% of 29 GPOH-POCs were able to perform a quantitative analysis of blood cultures or a *differential time to positivity* in their in-house or external microbiology department.

The DTP measures the time from blood culture sampling to the first positive signal indicating growth in a blood culture bottle. If a difference in time to detection of more than 2 hours is automatically documented (blood culture from the CVAD positive at least 2 hours earlier than from the peripheral venous cultures), the origin of the BSI may be the CVAD [99]. Chen et al. investigated the DTP method in children and adults with cancer [100], finding that the sensitivity of the DTP was 83% concerning those infections in which the origin of the BSI was the CVAD (catheter-related BSI; CR-BSI).

Handrup et al. from Aarhus (Denmark) examined 654 paired blood cultures between April 2008 and December 2012 in pediatric cancer patients with fever. The authors ultimately detected 112 BSI (17% of all febrile events). Of these, 64 (57%) were allocated to the category of CR-BSI, indicating that in 43% of all BSI, the CVAD was probably not the source of the BSI [89].

The validity of the DTP method depends on many critical control points. For instance, the CVAD must allow the necessary amount of blood to be drawn, and it is essential that this be the same volume per bottle as the peripheral venous blood culture. Furthermore, the time of sampling must be documented correctly; the storage and transport of the samples must be identical. Not all results of the DTP are discriminative (more than 2 h difference). In contrast to a quantitative examination of the blood cultures’ yield, the DTP is quite easily conducted in the microbiology laboratory. All things considered, the DTP appears to be the most feasible method to identify or exclude the CVAD as the source of the BSI without removal of the device. 

Certain clinical information may indicate that the CVAD is the most probable source of the BSI:

- Fever appears soon after flushing the CVAD;
- Subsequent blood cultures drawn from the CVAD remain positive for the same pathogen despite antibiotic treatment:
- Fever disappears after an ethanol-lock [51], [101] or immediately after removal of the CVAD.

## Different types of BSI in pediatric cancer patients

### CVAD-associated versus CVAD-related BSI (CA- vs. CR-BSI)

If the CVAD has been confirmed as the source of the BSI, the BSI is categorized as CR-BSI (**c**atheter **r**elated-BSI). Since this is difficult to prove in clinical practice (see above), many BSI in pediatric cancer patients without an identified alternative focus of bacteremia match the definition criteria for CVAD-associated BSIs (**c**atheter **a**ssociated-BSI; any positive blood culture in a symptomatic patient with a CVAD in use without another identifiable focus). Anglo-American surveillance systems use the term “central line-associated bloodstream infection (CLABSI)” to describe this category. In pediatric cancer patients with fever and neutropenia (with or without mucositis), it remains a challenge to detect the definite source of the BSI clinically. Even in well-trained surveillance personnel, this leads to uncertainties in how to correctly categorize a BSI, in particular when the attending physicians’ assessment classifies the BSI as secondary (related to a clinically undefined focus or to translocation from the mucous membranes of the patient) [102]. The **CA-BSI** definition aims for the almost complete detection and documentation of all BSIs (high sensitivity). 

On the other hand, a substantial proportion of all CA-BSI are not *related* to the CVAD (low specificity) [87] in this particular patient population [103], [104]. Based on these it is legitimate to question why it still makes sense to use the CA-BSI category in pediatric cancer patients.

In the recently published surveillance study by Ammann et al. [6], the proportion of CVAD removal in CA-BSIs was identical to the corresponding proportion in secondary BSIs (5% and 4%, respectively). In contrast, 26% of all CR-BSIs eventually resulted in the decision of the attending pediatric oncologist to remove the device.

If the infection control personnel only relies on the current definition of CA-BSI, this will interfere with the clinical practice and assessment of the attending pediatric oncologists [105], [104]. In addition, these BSI categorization problems have led to a plethora of different definitions [106] and to the suggestion to develop more specific, uniform definitions for this particular clinical setting [107]. 

The validity of the surveillance results becomes questionable when a significant proportion of all BSI is denominated *CVAD-associated*, although these BSIs are *not related* to the CVAD. Many CVAD-*associated* BSI cannot be prevented by increasing the clinical implementation of and compliance with preventive CVAD maintenance-care bundles [108]. This uncertainty may be one reason why only 42% of all German POCs included in a 2013 survey performed a prospective surveillance of BSI [19].

### Community- vs. nosocomially acquired BSI 

One dubious strategy to “decrease” BSI rates (adjusted to utilization days of the CVAD) in pediatric cancer patients is to differentiate community- and nosocomially acquired BSI and to exclude all “community-acquired BSI” from the final analysis of the surveillance data. 

Neither the CDC definitions nor the definitions of the German National Reference Center for the Surveillance of Nosocomial Infection (NRZ, Charité Berlin) offer a definite time frame within which to assign the BSI to one of the above categories. US-American authors [109], [110], [111] defined a latency of 48 hours (before or after admission to the hospital). The specificity of such an agreement is questionable in pediatric cancer patients, who often alternate between inpatient and outpatient treatment even during intensive chemotherapy periods [10], [11], [109], [111]. It is impossible to definitely determine when the contamination/colonization of the CVAD, which eventually leads to a CR-BSI in some but not all patients, has taken place.

Even in the outpatient setting, many manipulations of the CVAD hub are necessarily related to medical interventions, such as drawing blood for laboratory tests, outpatient chemotherapy, or transfusion of erythrocyte or thrombocyte concentrates. 

In the German Infection Protection Act, all infections related to a medical intervention are considered nosocomial [62], [112].

On the other hand, the relative risk of a CR-BSI is much higher in the inpatient setting. Two studies in pediatric cancer patients identified a risk ratio of about 8 compared to the outpatient setting [2], [9]; this seems plausible since inpatients require many more manipulations of the CVAD and the infusion system, and the proportion of patients with severe neutropenia and mucositis requiring morphine infusion and parenteral nutrition is much higher in inpatients. Referring to the above-mentioned definition, Rinke et al. compared all BSI in 319 pediatric oncology patients during a 22-month surveillance [111]. The authors detected 55 community-acquired CA-BSI (infection rate, IR, 0.65/1000 utilization days; CI95 0.49–0.85) and 19 inpatient CA-BSIs (infection rate 2.2, CI95 1.3–3.4). Of the patients with community-acquired CA-BSI, 13% had to be admitted to the PICU, and in 44%, the CVAD was removed during the course of the infection. As in many previous studies, the relative risk of a BSI event was significantly greater with Broviac/Hickman CVADS vs. Ports (odds ratio 20.6; CI95 7.6–69; p<0.001). Further independent risk factors were bone marrow transplantation in the preceding 10 days (odds ratio OR 16, CI95 1.1–264), medical history of a previous CA-BSI (OR 10, CI95 2.5–43), and CVAD implantation less than 4 weeks before the event (OR 4.2; CI95 1.0–17). Although the outpatient infection rate was lower (due to the greater number of “utilization days” in the denominator; RR 3.4; 0.65 vs. 2.2 CA-BSI per 1000 utilization days), the absolute number of events was 2.9-times higher in the outpatient setting (n=55 vs. n=19). Allen et al. previously came to the same conclusion (n=41 in outpatients vs. n=17 in inpatients) [9]. The recent Oncoped Surveillance study used 72 hours as an arbitrary time latency to assign the CA-BSI to the category “community-acquired”; this was the case in 43% of all documented BSIs [6]. 

The results of several recent studies demonstrate [109], [111], [9], [6] that the surveillance of BSI in pediatric cancer patients should not focus only on the inpatient setting, since this excludes important parts of the problem (epidemiology, risk profiles, pathogen distribution, in vitro resistance, clinical course of and resource allocation to “community-acquired BSIs”). The maintenance care of CVADs in POC outpatient clinics may increase the risk of CR-BSIs [113]. This may be particularly relevant if relatives/caregivers of the pediatric cancer patients are actively involved in maintenance care of the CVAD or manage these issues during home parenteral nutrition [114], [115].

### Mucosal barrier injury-associated bloodstream infection

To manage the uncertainties and challenges described above, in particular in patients with high risk of translocation [severe and protracted neutropenia, mucositis and/or graft-versus-host disease (GVHD)] [29], a consensus working group in the USA has recently developed a new category/definition: “Mucosal Barrier Injury Laboratory-Confirmed Bloodstream Infection” (MBI-LCBI). Referring to the unique epidemiology of BSI in patients with severe and protracted neutropenia, mucositis and/or GVHD [30], oncologists, infectious disease physicians, and infection control specialists (surveillance) piloted criteria for such a definition [116] and evaluated their feasibility and applicability in a study including 38 oncology centers [117]. The new MBI-LCBI category was implemented after minor modification [118]. The pilot study elucidated certain limitations of the new definition [117]. First, in many oncology centers, a differential leucocyte count was not available when the total number of leukocytes fell below a certain cut-off (e.g., 0.3x10^9^/L) [119]. In this regard, the criterion of neutropenia was adjusted to <0.5x10^9^/L (with a duration of at least 2 days temporally related to the BSI) [117]. Interestingly, in a recent Delphi study performed with a consortium of 45 international experts, no consensus was reached how to define *neutropenia* in the clinical context of fever in pediatric cancer patients [120]. 

The second obstacle in the above-mentioned pilot trial was the documentation of symptoms in the patient file, which may allow the grading of any GVHD of the gastrointestinal tract (in particular, number of bowel movements and quantification of volume losses related to diarrhea). These items were incompletely documented in 55% of the patients’ files. In 47%, grading of the GVHD was available in writing as case note derived from the attending oncologists [121]. In addition, the question remains as to which grading system this documentation of GVHD should finally refer to. 

Clinical studies often use the Common Terminology Criteria for Adverse Events (CTCAE; http://evs.nci.nih.gov/ftp1/CTCAE/About.html). The optimal/most feasible grading system for GVHD is still a matter of debate among oncologists performing allogeneic bone marrow or stem cell transplants [122]. Severe GVHD, with fluid loss of more than 1 liter per day or more than 20ml/kg bodyweight in children, leads to medical intervention intended to reduce fluid- and electrolyte losses in patients with GVHD-related diarrhea. Different medical intervention strategies certainly have a variable influence on the GVHD severity (risk of confounding).

Finally, the new MBI-CLBSI definition has worked as a self-fulfilling prophecy, leading to a significant reduction of CA-BSI in oncology patients as a result of a change in categorization. In the study performed by See et al. [117], this was the case in 37% of all BSI (45% in 10 participating specialized oncology treatment centers, including 2 POCS). In 91%, neutropenia was the leading criterion (GVHD only in 9%). The study by Metzger et al. [118] describes a more pronounced consequence of the new definition: of 66 BSIs without a clinically or microbiologically defined secondary focus of infection, 47 (71%) were allocated to the new MBI-LCBI category; only 19 (29%) remained in the traditional CA-BSI category. As in the See study, neutropenia was the leading criterion; only 9% of all cases in the MBI-LCBI group displayed GVHD (“any grade”), and only 40% mucositis (“any grade”). The most prevalent pathogens in MBI-LCBIs were *E. coli* (32%), *Enterococcus faecium* (30%) and VGS (21%), in contrast to *S. aureus* (26%), CoNS (21%), and *P. aeruginosa* (16%) in the remaining cases. How did this new categorization influence infection rates? Without the new definition of MBI-LCBI, the IR was 3.21 BSI/1000 utilization days, but after implementation of the MBI-LCBI definition, the IR was much lower (0.6). In this regard, the new definition led to a relative reduction of the IR by a factor of 5.4 (only 6 of 32 events were defined as CA-BSI). Surprisingly, 47% of the non-MBI-LCBI patients had mucositis (“any grade”), and in both groups, the majority of the central venous catheters were removed related to the infection (MBI-LCBI group, 64%; non-MBI-LCBI group, 74%).

No patient in the non-MBI-LCBI group died related to the BSI, but 15% of all patients in the MBI-LCBI group did, temporally related to the BSI (p=0.18). In 74% of the non-MBI-LCBI patients with granulocytopenia (74%), the isolated pathogen did not allow allocation to the MBI-LCBI group (only certain species are allowed).

At best, the introduction and practical implementation of this new BSI category in the CDC definition can be described as a “work in progress” [119]; unfortunately, it appears that one unspecific definition (CA-BSI) has been replaced by another (MBI-LCBI), which still leaves a great deal of room for individual interpretation [102], [123]. In addition, it is not known how or whether the incidence of MBI-LCBI is influenced by CVAD maintenance care bundles [118], although it remains probable that some of these BSI stem from the CVAD.

This assumption was proven very impressively in a recent study by Shelburne et al. [31]. Those authors retrospectively analyzed BSI caused by VGS (n=82). In these febrile adult oncology patients, central venous and peripheral venous cultures were drawn and quantitatively analyzed. The BSI was attributed to the CVAD when the quantitative analysis revealed a ≥3x higher CFU count in the central venous culture or when a semiquantiative culture of the catheter tip after removal showed growth of more than 15 CFU [93]. Following this diagnostic stratification, 27 of 82 VGS BSI (33%) were categorized as CR-BSI. Patients with such an event were significantly more often neutropenic and had significantly more often received FCH prophylaxis (in this group, the prevalence of FCH-resistant VGS reached 81%, as opposed to 54% in those patients without FCH prophylaxis). In the CR-BSI group, the central venous catheter was significantly more often removed during the infection (22% vs. 2%). If the new MBI-LCBI criteria had been applied, 71 (87%) would have been assigned to this category, in most cases due to the concomitant items neutropenia and BSI due to VGS [31].

### Pay for Performance as an important reason for changing the surveillance definitions in the U.S.A

In the U.S.A., the surveillance definitions for healthcare-associated infections are determined by the Centers for Disease Control and Prevention (CDC) and the National Health and Safety Network (NHSN) [87]. Since 2011, some health care authorities/regulation agencies have regional recommendations to publish infections rates on public websites (public reporting) [124]. This decision was prompted by the assumption that most of these BSI are preventable events [125], [126]. Hospitals with continuously high IRs should realize that public reporting may result in a negative medical reputation and are requested to increase their efforts in terms of prevention [127]. In addition, some of the most important reimbursement institutions, such as the Centers for Medicare and Medicaid Services (http://medicaid.gov/), refuse to pay for this “bad performance” (preventable complications). This pay-for-performance concept coupled with zero tolerance towards preventable BSIs compels the hospital administration to invest in prevention [128]. 

Such a legal and financial framework places substantial pressure on those institutions which define the corresponding events; the question “*Is this BSI really a CR-BSI?*” gains paramount importance [105]. Physicians who care for high-risk oncology patients may experience a conflict of interest, and the official allocation of a BSI event to the CA-BSI or CR-BSI category will become less probable. Public benchmarking between hospitals exacerbates this conflict [129], [130]. Fortunately, there is hitherto no recommendation for public reporting and benchmarking of BSI infection rates in Germany, Austria, or Switzerland (in Germany, there is only a notification requirement for BSI caused by methicillin-resistant *S. aureus*).

## Preventive bundle studies to prevent CR-BSI

Prospective surveillance of BSIs and regular feedback of infection rates are not ends in themselves; following the primary assumptions of the infection protection act, data derived from prospective surveillance is necessary to support the preventive efforts of the treatment team. The prevention of BSI is one of the most important means of infection prevention in pediatric cancer patients, and optimizes patient safety, quality of medical treatment, and reasonable allocation of limited resources [18], [109], [110], [131], [132]. One step in the right direction is to implement preventive maintenance-care bundles for CVADs.

Table 2 (Tab. 2) summarizes maintenance-care bundle studies performed in POCs and published by the end of 2015. In contrast to corresponding efforts in neonatal or pediatric intensive care units [133], [15], the most important components of prevention do not concern the insertion/implantation [134] but maintenance care of the CVAD [46], [132], since the implantation of the CVAD is performed in a pediatric surgical theater accompanied by perioperative antibiotic prophylaxis (in some POCs).

Examples for critical control points of CVAD maintenance care to increase patient safety [18] are:

- Hand disinfection before any manipulation of the CVAD hub or the infusion system [135];
- The use of antiseptics containing octenidine or chlorhexidine for local care of the catheter exit site, as local antiseptic before transcutaneous access to a fully implanted port, and on the catheter hub/on needle-free connecting devices or three-way stopcocks before and after each manipulation;
- Strict aseptic approach when changing the dressing at the Broviac exit site;
- Flushing of the Broviac catheter only once or twice per week if not in use; no flushing of ports which are not in use (but locked with heparin 100 U/ml or a lock solution containing taurolidin);
- The use of ready-to-use flushing syringes with 10 ml sterile 0.9% sodium chloride solution;
- The reduction of the frequency of routine IV system changes to at least 96 hours [exceptions: lipid-containing parenteral nutrition (24 hours) or after blood transfusion (8 hours)]
- Education and training in, as well as supervision of any aseptic reconstitution of IV medication following a written, internal standard operating procedure;
- In case of sustained high infection rates: use of chlorhexidine-releasing dressings at the entry site of the Broviac/Hickman [136], [137] or use of lock solutions which possess antimicrobial activity [138], [101].

It remains an unresolved issue whether the routine use of chlorhexidine-containing washcloths [91], [132], [139], [140], [141] or octenidine-containing shower gels (formerly used for MRSA decolonization [142], [143]) provides an additional benefit in terms of BSI prevention in pediatric cancer patients outside the acute post-bone-marrow or -stem-cell transplantation setting [91], [132].

The implementation of prevention bundles may depend on some changes in clinical practice and culture:

- Some healthcare workers and physicians may have to change their individual perspective on BSI (BSIs are not a matter of fate, but in many cases a preventable complication within the scope of our clinical responsibilities);
- Strategies for maintenance care have to be defined following an interdisciplinary approach involving all frontline personnel and perhaps some of the parents/caregivers;
- Correct handling should be practiced on a dummy before being performed without supervision by medical personnel;
- Implementation must be accompanied and followed by quality assurance efforts (audits, plan-do-check-act cycles) [144], [145],
- Local leaders have to accept and exercise their outstanding responsibility [146].

It is very important to guarantee timely feedback on obstacles and circumstances which hinder the practical implementation of the prevention bundle. In addition, frontline personnel should receive regular feedback on the effects of this intervention.

### Problems regarding the statistical significance of prevention studies

In most German POCS, the number of admitted patients is too low to reach statistical significance in terms of reduced infection rates in a monocentric epidemiological study, even when adjusted statistical methods, such as interrupted time series analysis, are used and the observation period in each group is longer than 24 months [147], [110]. Referring to monocentric results from Homburg/Germany [148], more than 400 consecutive pediatric cancer patients would have to be included to demonstrate a significant benefit (power of 80%, two-sided testing, p<0.05).

Although such a large number of included patients may be generated in multicenter studies (e.g., involving all interested GPOH pediatric oncology centers), an identical prevention bundle must be defined for all participating POCs [19], [149].

## How to move on

Considering the previously discussed framework of information regarding the surveillance of BSI in POCs, the pediatric oncologists among the current authors suggest some hallmarks for the conception of a standard surveillance module (registry) for BSI in POCs. It is important to emphasize that the discussion of the related issues with our colleagues from the NRZ for the Surveillance of Nosocomial Infection (Charité, Berlin) is an ongoing process. 

The following suggestions were made by pediatric oncologists, some of whom have been principal investigators or local coordinators of previous surveillance studies in this field. The issue at stake is to determine which data are needed from the perspective of the attending physicians

- to investigate the effectiveness and safety of the local preventive strategy (maintenance care bundle) in the long term;
- to fulfil the documentation requirements of the German Infection Prevention Act (§ 23; no public reporting but obligatory internal documentation) [112] considering
  a) infection rates related to the use of CVADS and
  b) local epidemiology (pathogens and resistance profiles derived from blood culture analysis) [62].

The resources (personnel and time) for these efforts are limited; a realistic exploration of the current situation leads to the conclusion that the items included in such a surveillance module must be much more restricted than those necessary for a scientific study.

### Requirements for organizational structure

Participation of POCS in the new surveillance module should remain voluntary, although the managing board of the GPOH will definitely endorse participation. In the near future, participation in the new surveillance module will become an item of quality assurance in POCs. 

The medical director and the hospital administration of the POC have to approve and declare participation in the new module in advance. The local coordinators should perform an upfront analysis concerning the personnel and time resources necessary for its active implementation. 

The results of local surveillance are primarily used for long-term quality assurance and infection prevention in the participating POC. In any benchmarking presentation of results comparing data of different POCs (national reference data), the location from which the results have been retrieved will be anonymized. Infection rates of individual POCS will be handled confidentially within the cooperative multicenter surveillance group. 

All participating centers provide the central coordinators with basic epidemiological information considering their POC, such as the absolute number of newly admitted pediatric cancer patients per year, number of patients with relapsed malignancy per year, number of autologous stem cell transplantations, and details of the local strategy of BSI prevention.

### Inclusion criteria

All pediatric cancer patients up to 21 years of age will be included anonymously if they have a CVAD in use and are being treated with conventional chemotherapy, radiation therapy, or high dose chemotherapy with autologous stem cell transplantation.

### Diagnostic blood culture sampling

The local coordinators of the surveillance initiative will receive a Word file, which may be used as a template to adjust the local guideline concerning blood culture sampling in pediatric cancer patients with fever (with or without concomitant neutropenia). The purpose of this document is to guarantee a minimum of standardization, e.g., considering the minimal volume of blood sampling for blood cultures depending on body weight of the patient. The participating POCS should have an internal, written consensus protocol for the sampling and processing of blood cultures. Details of this protocol are left at the discretion of the local coordinators and the respective microbiology laboratory.

### Which BSI should be documented?

The surveillance documents all blood-culture-positive (laboratory confirmed) BSI in symptomatic patients. The basic list of all events should be matched with the monthly microbiological laboratory results [150]. All BSI are included, in which the attending physicians do not attribute the positive yield to contamination. In case of a primary focus at any other site considered as the probable focus of the BSI by the attending pediatric oncologists, the BSI is marked as secondary. The primary focus should be documented in the case report form of the module.

Even if only one positive blood culture set was taken before antibiotic treatment was implemented, the clinical assessment of the attending physicians may allow the inclusion of the event. In this regard, it must be borne in mind that opportunistic pathogens such as CoNS, VGS and enterococci represent real pathogens in pediatric cancer patients. The probability of a true-positive result is higher when the pathogen grows in both bottles (aerobic and anaerobic) with a positive yield in the first 48 hours after sampling.

All BSI are included independent of the setting in which the patients developed initial symptoms (as an out- or in-patient). Although this time latency remains arbitrary, BSIs may be allocated to the category “acquired during inpatient treatment” if the first symptoms occur 72 hours after admission or less than 72 hours after the patient has left the hospital [6]. This allows for the normalization of inpatient BSIs to 1000 inpatient treatment days (incidence density = events / inpatient treatment days x 1000). Monthly inpatient treatment days can easily be obtained from hospital administration.

### Reporting microbiology results

The results of blood culture diagnostics should be reported by the microbiology laboratory as follows:

- Detected pathogens on a species level (max. 3 per blood culture) [*The definite documentation of polymicrobial BSI requires a consensus in the coordination group.*];
- Specific in vitro resistance and multidrug resistance [62], [63] including penicillin G resistance in VGS, MRSA, VRE, 2 MRGN NeoPäd [151], 3 and 4 MRGN [63], [74].

In POCs, in which FCH are routinely used for prophylaxis or treatment, FCH resistance (cipro- and levofloxacin) in Gram-negative pathogens should be additionally reported.

### Additional clinical items reported in the CRF

Additional items which may be reported in the CRF (related to a particular BSI event) are a local identification code for the patient (second or third BSI?), age of the patient in years/months, gender, underlying malignancy, first illness or relapse, treatment protocol (GPOH), treatment with antibiotics (therapeutically or prophylactically, except cotrimoxazol prophylaxis), type of CVAD (Broviac or Port), home parenteral nutrition, local infection at the CVAD entry site (or port pocket), probable source of the BSI in secondary cases, presence of neutropenia (<0.5x10^9^/L or leukocytes <1x10^9^/L and no differential cell count available ±3 days from the event), mucositis which results in morphine infusion or parenteral nutrition, any clinically significant GVHD of the mucous membranes, anorectal infiltration, and *Ecthyma gangraenosum* [152], [153].

Further items describing the clinical course of the event may be documented:

- Clinical severity (bacteremia, sepsis, septic shock, septic shock with multiorgan failure) [25], [6], [1];
- Adjuvant use of ethanol-lock therapy [101] (or any other antimicrobial lock therapy) [154];
- Removal of the CVAD related to the BSI;
- Outcome: recovery, palliative care or death of the patient (related to the BSI);
- Inpatient treatment days and intensive care treatment days related to the BSI;
- Duration of antibiotic treatment (days of antibiotics related to the BSI);
- Duration of neutropenia after the onset of treatment (if documented in the patient’s file).

Items to be documented optionally

- Empirical first-line treatment of the BSI (e.g. piperacillin-tazobactam ± aminoglycoside, ceftazidime, meropenem, etc.);
- Targeted therapy (after the pathogen and its in vitro sensitivity is available);
- Concomitant antifungal treatment (amphotericin B, caspofungin, voriconazole, etc.).

### Responsibility, accountability, leadership and necessary resources

In Germany, the medical directors of the POC are responsible for the implementation of prospective surveillance and preventive strategies according to the Infection Prevention Act (IfSG). In general, the medical directors have a profound interest in guaranteeing patient safety in their unit, and in the avoidance of preventable medical complications. The same should valid for hospital administration, accountable for personnel allocation, purchasing all necessary materials and medical products, and quality management.

Without the personal investment of the POC leaders [155], [146], such a surveillance initiative cannot be implemented sustainably (leadership and clinical culture promoting patient safety are mandatory) [156], [157]. The prospective surveillance of BSI is paramount to the quality management in POCS, and sampling the primary data is the responsibility of the local infection control personnel [158]. The infection control personnel should be supported by members of the pediatric oncology treatment team. The necessary personnel and time resources must be prospectively provided by hospital administration. The time required to educate new team members in the details of the surveillance module must also be taken into account.

It is of utmost importance to inform the whole treatment team (frontline physicians and healthcare workers) about the aims, methods, and scope of the surveillance initiative. The sampling of primary data is much more efficient if these items are clearly documented in the patient’s file. The completion of any electronic case report form needs confirmation by the infection control personnel and a pediatric oncologist.

### Analysis and feedback

If such a surveillance module is available on a protected internet platform and all basic data (such as inpatient treatment days) have been provided by the local coordinators, it allows timely, automatic reporting of cumulative results. A standard format for these reports should be developed, which contains information on the bacterial species spectrum and on specific resistance profiles in the most prevalent pathogens (see above) (§ 23 IfSG) [62], [159]. In most centers, such feedback should be generated every 6 months. The report should detail the results month by month in a clearly arranged format. These results are not generated for confidential storage but should be regularly discussed with the treatment team [112], the microbiologists, and the infection control personnel. In addition, the establishment of a multicenter surveillance consortium will facilitate the discussion of the results between the local coordinators of the participating POCs. All issues related to this important topic should be discussed at least once a year in a central meeting of the local study coordinators.

### BSI rates and patient-to-nurse ratios

Since there may be a relationship between the number of available, well-qualified healthcare workers (HCWs) and BSI rates, the general assembly of the GPOH in Berlin (Charité, May 30, 2015) suggested documenting the number of available pediatric oncology HCWs in the module. One feasible method is the daily documentation of the number of inpatients and the number of available HCWs as a patient-to-nurse ratio (inpatients : HCWs) [160]. One obstacle is that many centers provide care for patients during the day (e.g., diagnostic procedures with analgosedation, blood transfusion, chemotherapy), but these patients – although on the ward during daylight hours – do not appear in the midnight census. This issue should be discussed and decided in the surveillance coordination group.

## Conception and ethical framework of quality management initiatives

Quality management (QM) and quality improvement initiatives in healthcare facilities comprise a specific approach of experimental learning, with sustained development and implementation of new standards of care and defined workflows as central elements of clinical practice [145], [161]. The particular instruments and strategies involved in QM initiatives are chosen by the treatment team after a thorough examination of the available evidence and experience, assuming that these strategies will result in a significant benefit for the patients and the hospital. These initiatives depend on the sampling of structured data documented in the patients’ files during routine clinical workflows.

Patients and their families/caregivers are vitally interested in receiving the best available treatment; this implies consent with clinical initiatives implemented systematically to improve patient safety [162]. In contrast to controlled scientific studies comparing different interventions, for which written informed consent of the patients and/or their parents/caregivers is obligatory, QM initiatives feature other points:

- QM initiatives refer to a clinical standard of care which is executed in all patients;
- QM initiatives only rely on clinical routine data available in the patients’ files.
- QM initiatives do not contain a specific intervention associated with or suspected to display an increased risk of any medical complication.
- The protection of personal data privacy is guaranteed, since the central data analysis uses anonymized patient data, although the sampling of the primary data necessitates confidential access of qualified medical personnel to the patients’ files.
- The primary goal of QM is improvement of the quality of medical care and safety in the participating institution.

The prospective surveillance of BSIs in pediatric cancer patients to investigate the effects of a preventive maintenance care bundle is a concrete example for such a QM initiative. 

The suggestions presented here, derived from a comprehensive review of the available evidence and experience in this field, set the stage for a new surveillance module adjusted to § 23 of the German Infection Prevention Act [112]. The IfSG and its translation and integration into the hospital hygiene regulations of the different federal states in Germany constitute a legal framework for local [163], [164] and multicenter QM projects aiming at the reduction of BSI in pediatric cancer patients [165]. It appears reasonable to inform the patients and their caregivers upon their first hospital admission that the POC takes part in such a QM initiative in order to continually improve clinical practice.

## Notes

### Competing interests

The authors declare that they have no competing interests.

## Figures and Tables

**Table 1:**
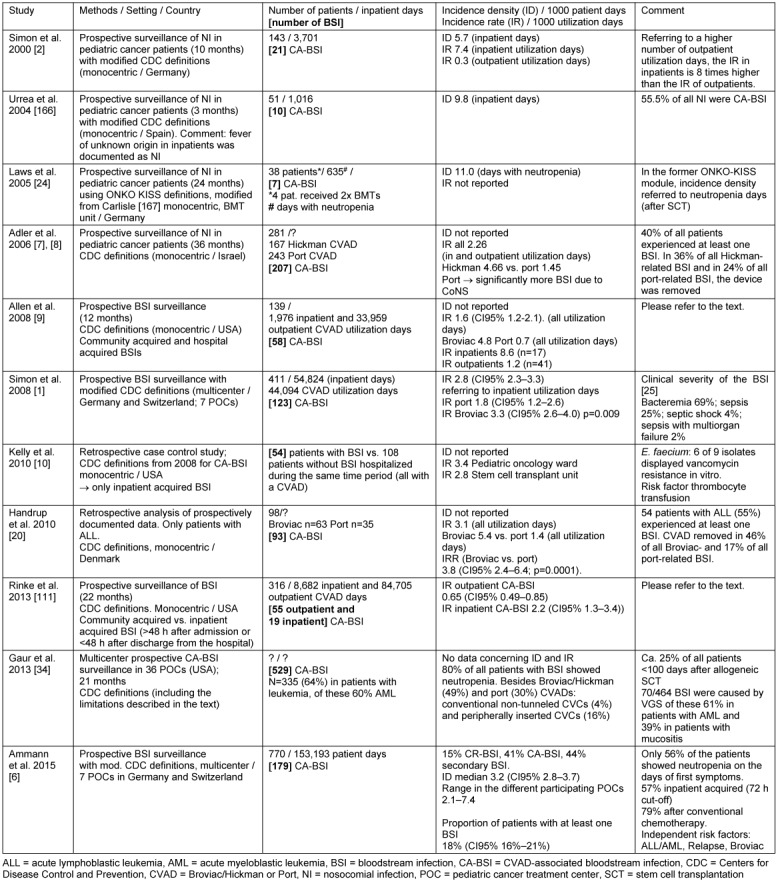
Surveillance of bloodstream infections in pediatric cancer patients (studies selected by the present authors)

**Table 2:**
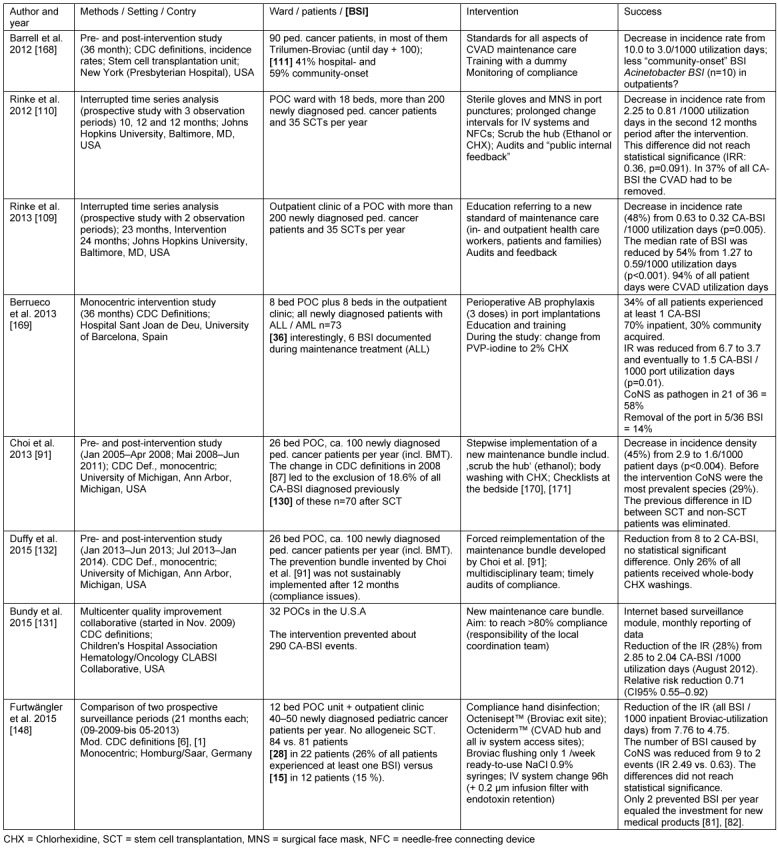
Preventive bundle studies (prevention of BSI in pediatric cancer patients)

## References

[1] Simon A, Ammann RA, Bode U, Fleischhack G, Wenchel HM, Schwamborn D, Gravou C, Schlegel PG, Rutkowski S, Dannenberg C, Körholz D, Laws HJ, Kramer MH (2008). Healthcare-associated infections in pediatric cancer patients: results of a prospective surveillance study from university hospitals in Germany and Switzerland. BMC Infect Dis.

[2] Simon A, Fleischhack G, Hasan C, Bode U, Engelhart S, Kramer MH (2000). Surveillance for nosocomial and central line-related infections among pediatric hematology-oncology patients. Infect Control Hosp Epidemiol.

[3] Miedema KG, Winter RH, Ammann RA, Droz S, Spanjaard L, de Bont ES, Kamps WA, van de Wetering MD, Tissing WJ (2013). Bacteria causing bacteremia in pediatric cancer patients presenting with febrile neutropenia--species distribution and susceptibility patterns. Support Care Cancer.

[4] Ammann RA, Bodmer N, Hirt A, Niggli FK, Nadal D, Simon A, Ozsahin H, Kontny U, Kühne T, Popovic MB, Lüthy AR, Aebi C (2010). Predicting adverse events in children with fever and chemotherapy-induced neutropenia: the prospective multicenter SPOG 2003 FN study. J Clin Oncol.

[5] Ammann RA, Hirt A, Lüthy AR, Aebi C (2004). Predicting bacteremia in children with fever and chemotherapy-induced neutropenia. Pediatr Infect Dis J.

[6] Ammann RA, Laws HJ, Schrey D, Ehlert K, Moser O, Dilloo D, Bode U, Wawer A, Schrauder A, Cario G, Laengler A, Graf N, Furtwängler R, Simon A (2015). Bloodstream infection in paediatric cancer centres--leukaemia and relapsed malignancies are independent risk factors. Eur J Pediatr.

[7] Adler A, Yaniv I, Solter E, Freud E, Samra Z, Stein J, Fisher S, Levy I (2006). Catheter-associated bloodstream infections in pediatric hematology-oncology patients: factors associated with catheter removal and recurrence. J Pediatr Hematol Oncol.

[8] Adler A, Yaniv I, Steinberg R, Solter E, Samra Z, Stein J, Levy I (2006). Infectious complications of implantable ports and Hickman catheters in paediatric haematology-oncology patients. J Hosp Infect.

[9] Allen RC, Holdsworth MT, Johnson CA, Chavez CM, Heideman RL, Overturf G, Lemon D, Hunt WC, Winter SS (2008). Risk determinants for catheter-associated blood stream infections in children and young adults with cancer. Pediatr Blood Cancer.

[10] Kelly M, Conway M, Wirth K, Potter-Bynoe G, Billett AL, Sandora TJ (2011). Moving CLABSI prevention beyond the intensive care unit: risk factors in pediatric oncology patients. Infect Control Hosp Epidemiol.

[11] Kelly MJ, Vivier PM, Panken TM, Schwartz CL (2010). Bacteremia in febrile nonneutropenic pediatric oncology patients. Pediatr Blood Cancer.

[12] Raymond J, Aujard Y (2000). Nosocomial infections in pediatric patients: a European, multicenter prospective study. European Study Group. Infect Control Hosp Epidemiol.

[13] Niedner MF, 2008 National Association of Children's Hospitals and Related Institutions Pediatric Intensive Care Unit Patient Care FOCUS Group (2010). The harder you look, the more you find: Catheter-associated bloodstream infection surveillance variability. Am J Infect Control.

[14] Niedner MF, Huskins WC, Colantuoni E, Muschelli J, Harris JM, Rice TB, Brilli RJ, Miller MR (2011). Epidemiology of central line-associated bloodstream infections in the pediatric intensive care unit. Infect Control Hosp Epidemiol.

[15] Miller MR, Niedner MF, Huskins WC, Colantuoni E, Yenokyan G, Moss M, Rice TB, Ridling D, Campbell D, Brilli RJ, National Association of Children's Hospitals and Related Institutions Pediatric Intensive Care Unit Central Line-Associated Bloodstream Infection Quality Transformation Teams (2011). Reducing PICU central line-associated bloodstream infections: 3-year results. Pediatrics.

[16] Simon A, Exner M (2014). Prävention nosokomialer Infektionen bei intensivmedizinisch behandelten Frühgeborenen. Monatsschr Kinderheilkd.

[17] Costello JM, Graham DA, Morrow DF, Potter-Bynoe G, Sandora TJ, Laussen PC (2009). Risk factors for central line-associated bloodstream infection in a pediatric cardiac intensive care unit. Pediatr Crit Care Med.

[18] Simon A, Beutel K, Laws HJ, Trautmann M, Greiner J, Graf N (2013). Evidenzbasierte Empfehlungen zur Anwendung dauerhaft implantierter, zentralvenöser Zugänge in der pädiatrischen Onkologie.

[19] Simon A, Graf N, Furtwängler R (2013). Results of a multicentre survey evaluating clinical practice of port and Broviac management in paediatric oncology. Klin Padiatr.

[20] Handrup MM, Møller JK, Frydenberg M, Schrøder H (2010). Placing of tunneled central venous catheters prior to induction chemotherapy in children with acute lymphoblastic leukemia. Pediatr Blood Cancer.

[21] Kommission für Krankenhaushygiene und Infektionsprävention beim Robert Koch-Institut (RKI) (2010). Anforderungen an die Hygiene bei der medizinischen Versorgung von immunsupprimierten Patienten. Empfehlung der Kommission für Krankenhaushygiene und Infektionsprävention beim Robert Koch-Institut (RKI). Bundesgesundheitsblatt Gesundheitsforschung Gesundheitsschutz.

[22] Gorelick MH, Owen WC, Seibel NL, Reaman GH (1991). Lack of association between neutropenia and the incidence of bacteremia associated with indwelling central venous catheters in febrile pediatric cancer patients. Pediatr Infect Dis J.

[23] Dettenkofer M, Wenzler-Röttele S, Babikir R, Bertz H, Ebner W, Meyer E, Rüden H, Gastmeier P, Daschner FD, Hospital Infection Surveillance System for Patients with Hematologic/Oncologic Malignancies Study Group (2005). Surveillance of nosocomial sepsis and pneumonia in patients with a bone marrow or peripheral blood stem cell transplant: a multicenter project. Clin Infect Dis.

[24] Laws HJ, Kobbe G, Dilloo D, Dettenkofer M, Meisel R, Geisel R, Haas R, Göbel U, Schulze-Röbbecke R (2006). Surveillance of nosocomial infections in paediatric recipients of bone marrow or peripheral blood stem cell transplantation during neutropenia, compared with adult recipients. J Hosp Infect.

[25] Goldstein B, Giroir B, Randolph A, International Consensus Conference on Pediatric Sepsis (2005). International pediatric sepsis consensus conference: definitions for sepsis and organ dysfunction in pediatrics. Pediatr Crit Care Med.

[26] Haupt R, Romanengo M, Fears T, Viscoli C, Castagnola E (2001). Incidence of septicaemias and invasive mycoses in children undergoing treatment for solid tumours: a 12-year experience at a single Italian institution. Eur J Cancer.

[27] Blijlevens NM, Donnelly JP, De Pauw BE (2000). Mucosal barrier injury: biology, pathology, clinical counterparts and consequences of intensive treatment for haematological malignancy: an overview. Bone Marrow Transplant.

[28] van der Velden WJ, Herbers AH, Netea MG, Blijlevens NM (2014). Mucosal barrier injury, fever and infection in neutropenic patients with cancer: introducing the paradigm febrile mucositis. Br J Haematol.

[29] Samet A, Sledzinska A, Krawczyk B, Hellmann A, Nowicki S, Kur J, Nowicki B (2013). Leukemia and risk of recurrent Escherichia coli bacteremia: genotyping implicates E. coli translocation from the colon to the bloodstream. Eur J Clin Microbiol Infect Dis.

[30] Steinberg JP, Robichaux C, Tejedor SC, Reyes MD, Jacob JT (2013). Distribution of pathogens in central line-associated bloodstream infections among patients with and without neutropenia following chemotherapy: evidence for a proposed modification to the current surveillance definition. Infect Control Hosp Epidemiol.

[31] Shelburne SA, Chaftari AM, Jamal M, Al Wohoush I, Jiang Y, Abughazaleh S, Cairo J, Raad S, Debiane L, Raad I (2014). Identification and characterization of catheter-related bloodstream infections due to viridans group streptococci in patients with cancer. Am J Infect Control.

[32] Austin PD, Elia M (2009). A systematic review and meta-analysis of the risk of microbial contamination of aseptically prepared doses in different environments. J Pharm Pharm Sci.

[33] Caselli D, Petris MG, Rondelli R, Carraro F, Colombini A, Muggeo P, Ziino O, Melchionda F, Russo G, Pierani P, Soncini E, DeSantis R, Zanazzo G, Barone A, Cesaro S, Cellini M, Mura R, Milano GM, Meazza C, Cicalese MP, Tropia S, De Masi S, Castagnola E, Aricò M, Infectious Diseases Working Group of the Associazione Italiana Ematologia Oncologia Pediatrica (2014). Single-day trimethoprim/sulfamethoxazole prophylaxis for Pneumocystis pneumonia in children with cancer. J Pediatr.

[34] Gaur AH, Bundy DG, Gao C, Werner EJ, Billett AL, Hord JD, Siegel JD, Dickens D, Winkle C, Miller MR, Children's Hospital Association Hematology-Oncology Quality Transformation Collaborative Project (2013). Surveillance of hospital-acquired central line-associated bloodstream infections in pediatric hematology-oncology patients: lessons learned, challenges ahead. Infect Control Hosp Epidemiol.

[35] Levinsen M, Shabaneh D, Bohnstedt C, Harila-Saari A, Jonsson OG, Kanerva J, Lindblom A, Lund B, Andersen EW, Schmiegelow K, Nordic Society of Paediatric Haematology and Oncology (NOPHO) (2012). Pneumocystis jiroveci pneumonia prophylaxis during maintenance therapy influences methotrexate/6-mercaptopurine dosing but not event-free survival for childhood acute lymphoblastic leukemia. Eur J Haematol.

[36] Agrawal AK, Chang PP, Feusner J (2011). Twice weekly Pneumocystis jiroveci pneumonia prophylaxis with trimethoprim-sulfamethoxazole in pediatric patients with acute lymphoblastic leukemia. J Pediatr Hematol Oncol.

[37] Pyrgos V, Shoham S, Roilides E, Walsh TJ (2009). Pneumocystis pneumonia in children. Paediatr Respir Rev.

[38] Ohata Y, Ohta H, Hashii Y, Tokimasa S, Ozono K, Hara J (2009). Intermittent oral trimethoprim/sulfamethoxazole on two non-consecutive days per week is effective as Pneumocystis jiroveci pneumonia prophylaxis in pediatric patients receiving chemotherapy or hematopoietic stem cell transplantation. Pediatr Blood Cancer.

[39] Neumann S, Krause SW, Maschmeyer G, Schiel X, von Lilienfeld-Toal M, Infectious Diseases Working Party (AGIHO), German Society of Hematology and Oncology (DGHO) (2013). Primary prophylaxis of bacterial infections and Pneumocystis jirovecii pneumonia in patients with hematological malignancies and solid tumors : guidelines of the Infectious Diseases Working Party (AGIHO) of the German Society of Hematology and Oncology (DGHO). Ann Hematol.

[40] Hersh AL, Gerber JS, Hicks LA, Pavia AT (2015). Lessons Learned in Antibiotic Stewardship: Fluoroquinolone Use in Pediatrics. J Pediatric Infect Dis Soc.

[41] Castagnola E, Haupt R, Micozzi A, Caviglia I, Testi AM, Giona F, Parodi S, Girmenia C (2005). Differences in the proportions of fluoroquinolone-resistant Gram-negative bacteria isolated from bacteraemic children with cancer in two Italian centres. Clin Microbiol Infect.

[42] Sung L, Manji A, Beyene J, Dupuis LL, Alexander S, Phillips R, Lehrnbecher T (2012). Fluoroquinolones in children with fever and neutropenia: a systematic review of prospective trials. Pediatr Infect Dis J.

[43] Bradley JS, Jackson MA, Committee on Infectious Diseases, American Academy of Pediatrics (2011). The use of systemic and topical fluoroquinolones. Pediatrics.

[44] Paulus SC, van Saene HK, Hemsworth S, Hughes J, Ng A, Pizer BL (2005). A prospective study of septicaemia on a paediatric oncology unit: a three-year experience at The Royal Liverpool Children's Hospital, Alder Hey, UK. Eur J Cancer.

[45] Lehrnbecher T, Laws HJ, Boehm A, Dworzak M, Janssen G, Simon A, Groll AH (2008). Compliance with anti-infective preventive measures: A multicentre survey among paediatric oncology patients. Eur J Cancer.

[46] Krenn T, Fleischhack G, Moser O, Dilloo D, Bode U, Gräber S, Furtwängler R, Graf N, Simon A (2011). Bloodstream infections in paediatric cancer patients. Prospective comparative study in 2 university hospitals. Klin Padiatr.

[47] Kurt B, Flynn P, Shenep JL, Pounds S, Lensing S, Ribeiro RC, Pui CH, Razzouk BI, Rubnitz JE (2008). Prophylactic antibiotics reduce morbidity due to septicemia during intensive treatment for pediatric acute myeloid leukemia. Cancer.

[48] Inaba H, Gaur AH, Cao X, Flynn PM, Pounds SB, Avutu V, Marszal LN, Howard SC, Pui CH, Ribeiro RC, Hayden RT, Rubnitz JE (2014). Feasibility, efficacy, and adverse effects of outpatient antibacterial prophylaxis in children with acute myeloid leukemia. Cancer.

[49] Felsenstein S, Orgel E, Rushing T, Fu C, Hoffman JA (2015). Clinical and microbiologic outcomes of quinolone prophylaxis in children with acute myeloid leukemia. Pediatr Infect Dis J.

[50] Simon A, Gröger N, Wilkesmann A, Hasan C, Wiszniewsky G, Engelhart S, Kramer MH, Bode U, Ammann RA, Fleischhack G (2006). Restricted use of glycopeptides in paediatric cancer patients with fever and neutropenia. Int J Antimicrob Agents.

[51] Wolf J, Curtis N, Worth LJ, Flynn PM (2013). Central line-associated bloodstream infection in children: an update on treatment. Pediatr Infect Dis J.

[52] Doern CD, Burnham CA (2010). It's not easy being green: the viridans group streptococci, with a focus on pediatric clinical manifestations. J Clin Microbiol.

[53] Paulus S, Dobson S, Rassekh S, Blondel-Hill E (2009). In vitro inferiority of ceftazidime compared with other beta-lactams for viridans group Streptococcus bacteremia in pediatric oncology patients: implications for antibiotic choices. J Pediatr Hematol Oncol.

[54] Tunkel AR, Sepkowitz KA (2002). Infections caused by viridans streptococci in patients with neutropenia. Clin Infect Dis.

[55] Huang WT, Chang LY, Hsueh PR, Lu CY, Shao PL, Huang FY, Lee PI, Chen CM, Lee CY, Huang LM (2007). Clinical features and complications of viridans streptococci bloodstream infection in pediatric hemato-oncology patients. J Microbiol Immunol Infect.

[56] Lewis V, Yanofsky R, Mitchell D, Dix D, Ethier MC, Gillmeister B, Johnston D, Michon B, Stobart K, Portwine C, Silva M, Cellot S, Price V, Bowes L, Zelcer S, Brossard J, Beyene J, Sung L (2014). Predictors and outcomes of viridans group streptococcal infections in pediatric acute myeloid leukemia: from the Canadian infections in AML research group. Pediatr Infect Dis J.

[57] Johannsen KH, Handrup MM, Lausen B, Schrøder H, Hasle H (2013). High frequency of streptococcal bacteraemia during childhood AML therapy irrespective of dose of cytarabine. Pediatr Blood Cancer.

[58] Gassas A, Grant R, Richardson S, Dupuis LL, Doyle J, Allen U, Abla O, Sung L (2004). Predictors of viridans streptococcal shock syndrome in bacteremic children with cancer and stem-cell transplant recipients. J Clin Oncol.

[59] Schnappauf C, Rodloff A, Siekmeyer W, Hirsch W, Sorge I, Schuster V, Kiess W (2014). Invasive pneumococcal diseases in children and adolescents--a single centre experience. BMC Res Notes.

[60] Gomez B, Hernandez-Bou S, Garcia-Garcia JJ, Mintegi S, Bacteraemia Study Working Group from the Infectious Diseases Working Group, Spanish Society of Pediatric Emergencies (SEUP) (2015). Bacteremia in previously healthy children in emergency departments: clinical and microbiological characteristics and outcome. Eur J Clin Microbiol Infect Dis.

[61] Meisel R, Toschke AM, Heiligensetzer C, Dilloo D, Laws HJ, von Kries R (2007). Increased risk for invasive pneumococcal diseases in children with acute lymphoblastic leukaemia. Br J Haematol.

[62] Robert Koch-Institut (2013). Bekanntmachung des Robert Koch-Institutes: Surveillance nosokomialer Infektionen sowie die Erfassung von Krankheitserregern mit speziellen Resistenzen und Multiresistenzen. Fortschreibung der Liste der gemäß § 4 Abs. 2 Nr. 2 Buchstabe b in Verbindung mit § 23 Abs. 4 IfSG zu erfassenden nosokomialen Infektionen und Krankheitserreger mit speziellen Resistenzen und Multiresistenzen. Bundesgesundheitsblatt Gesundheitsforschung Gesundheitsschutz.

[63] Kommission für Krankenhaushygiene und Infektionsprävention beim Robert Koch-Institut (2011). Definition der Multiresistenz gegenüber Antibiotika bei gramnegativen Stäbchen im Hinblick auf Maßnahmen zur Vermeidung der Weiterverbreitung. Epidemiol Bulletin des Robert Koch-Instituts.

[64] Ewers C, Grobbel M, Stamm I, Kopp PA, Diehl I, Semmler T, Fruth A, Beutlich J, Guerra B, Wieler LH, Guenther S (2010). Emergence of human pandemic O25:H4-ST131 CTX-M-15 extended-spectrum-beta-lactamase-producing Escherichia coli among companion animals. J Antimicrob Chemother.

[65] Kola A, Kohler C, Pfeifer Y, Schwab F, Kühn K, Schulz K, Balau V, Breitbach K, Bast A, Witte W, Gastmeier P, Steinmetz I (2012). High prevalence of extended-spectrum-ß-lactamase-producing Enterobacteriaceae in organic and conventional retail chicken meat, Germany. J Antimicrob Chemother.

[66] Haeusler GM, Mechinaud F, Daley AJ, Starr M, Shann F, Connell TG, Bryant PA, Donath S, Curtis N (2013). Antibiotic-resistant Gram-negative bacteremia in pediatric oncology patients--risk factors and outcomes. Pediatr Infect Dis J.

[67] Simon A, Berger C, Bruns R, Heininger U, Deutsche Gesellschaft für pädiatrische Infektiologie (2013). Multiresistente Infektionserreger (Kapitel 5). DGPI Handbuch Infektionen bei Kindern und Jugendlichen.

[68] Arbeitsgruppe MRGN der Deutschen Gesellschaft für Pädiatrische Infektiologie und des Paed IC Projektes (2014). Infektionspräventives Vorgehen bei Nachweis von MRGN im Kindesalter. Hyg Med.

[69] Ariffin H, Navaratnam P, Mohamed M, Arasu A, Abdullah WA, Lee CL, Peng LH (2000). Ceftazidime-resistant Klebsiella pneumoniae bloodstream infection in children with febrile neutropenia. Int J Infect Dis.

[70] El-Mahallawy HA, El-Wakil M, Moneer MM, Shalaby L (2011). Antibiotic resistance is associated with longer bacteremic episodes and worse outcome in febrile neutropenic children with cancer. Pediatr Blood Cancer.

[71] Ciofi Degli Atti M, Bernaschi P, Carletti M, Luzzi I, García-Fernández A, Bertaina A, Sisto A, Locatelli F, Raponi M (2014). An outbreak of extremely drug-resistant Pseudomonas aeruginosa in a tertiary care pediatric hospital in Italy. BMC Infect Dis.

[72] Caselli D, Cesaro S, Ziino O, Zanazzo G, Manicone R, Livadiotti S, Cellini M, Frenos S, Milano GM, Cappelli B, Licciardello M, Beretta C, Aricò M, Castagnola E, Infection Study Group of the Associazione Italiana Ematologia Oncologia Pediatrica (AIEOP) (2010). Multidrug resistant Pseudomonas aeruginosa infection in children undergoing chemotherapy and hematopoietic stem cell transplantation. Haematologica.

[73] Tamma PD, Han JH, Rock C, Harris AD, Lautenbach E, Hsu AJ, Avdic E, Cosgrove SE, Antibacterial Resistance Leadership Group (2015). Carbapenem therapy is associated with improved survival compared with piperacillin-tazobactam for patients with extended-spectrum ß-lactamase bacteremia. Clin Infect Dis.

[74] Kommission für Krankenhaushygiene und Infektionsprävention beim Robert Koch-Institut (2012). Hygienemaßnahmen bei Infektionen oder Besiedlung mit multiresistenten gramnegativen Stäbchen. Empfehlung der Kommission für Kranken-haushygiene und Infektionsprävention (KRINKO) beim Robert Koch-Institut (RKI). Bundesgesundheitsblatt Gesundheitsforschung Gesundheitsschutz.

[75] Meckler G, Lindemulder S (2009). Fever and neutropenia in pediatric patients with cancer. Emerg Med Clin North Am.

[76] Lehrnbecher T, Phillips R, Alexander S, Alvaro F, Carlesse F, Fisher B, Hakim H, Santolaya M, Castagnola E, Davis BL, Dupuis LL, Gibson F, Groll AH, Gaur A, Gupta A, Kebudi R, Petrilli S, Steinbach WJ, Villarroel M, Zaoutis T, Sung L, International Pediatric Fever and Neutropenia Guideline Panel (2012). Guideline for the management of fever and neutropenia in children with cancer and/or undergoing hematopoietic stem-cell transplantation. J Clin Oncol.

[77] Averbuch D, Orasch C, Cordonnier C, Livermore DM, Mikulska M, Viscoli C, Gyssens IC, Kern WV, Klyasova G, Marchetti O, Engelhard D, Akova M, ECIL4, a joint venture of EBMT, EORTC, ICHS, ESGICH/ESCMID and ELN (2013). European guidelines for empirical antibacterial therapy for febrile neutropenic patients in the era of growing resistance: summary of the 2011 4th European Conference on Infections in Leukemia. Haematologica.

[78] Ammann RA, Tissing WJ, Phillips B (2012). Rationalizing the approach to children with fever in neutropenia. Curr Opin Infect Dis.

[79] Cheng S, Teuffel O, Ethier MC, Diorio C, Martino J, Mayo C, Regier D, Wing R, Alibhai SM, Sung L (2011). Health-related quality of life anticipated with different management strategies for paediatric febrile neutropaenia. Br J Cancer.

[80] Diorio C, Martino J, Boydell KM, Ethier MC, Mayo C, Wing R, Teuffel O, Sung L, Tomlinson D (2011). Parental perspectives on inpatient versus outpatient management of pediatric febrile neutropenia. J Pediatr Oncol Nurs.

[81] Biwersi C, Hepping N, Bode U, Fleischhack G, von Renesse A, Exner M, Engelhart S, Gieselmann B, Simon A (2009). Bloodstream infections in a German paediatric oncology unit: prolongation of inpatient treatment and additional costs. Int J Hyg Environ Health.

[82] Teuffel O, Amir E, Alibhai SM, Beyene J, Sung L (2011). Cost-effectiveness of outpatient management for febrile neutropenia in children with cancer. Pediatrics.

[83] Averbuch D, Cordonnier C, Livermore DM, Mikulska M, Orasch C, Viscoli C, Gyssens IC, Kern WV, Klyasova G, Marchetti O, Engelhard D, Akova M, ECIL4, a joint venture of EBMT, EORTC, ICHS, ESGICH/ESCMID and ELN (2013). Targeted therapy against multi-resistant bacteria in leukemic and hematopoietic stem cell transplant recipients: guidelines of the 4th European Conference on Infections in Leukemia (ECIL-4, 2011). Haematologica.

[84] Gyssens IC, Kern WV, Livermore DM, ECIL-4, a joint venture of EBMT, EORTC, ICHS and ESGICH of ESCMID (2013). The role of antibiotic stewardship in limiting antibacterial resistance among hematology patients. Haematologica.

[85] Lukac PJ, Bonomo RA, Logan LK (2015). Extended-spectrum ß-lactamase-producing Enterobacteriaceae in children: old foe, emerging threat. Clin Infect Dis.

[86] Weichert S, Simon A, von Müller L, Adam R, Schroten H (2015). Clostridium-difficile-assoziierte Infektionen im Kindes- und Jugendalter. Monatsschr Kinderheilkd.

[87] Horan TC, Andrus M, Dudeck MA (2008). CDC/NHSN surveillance definition of health care-associated infection and criteria for specific types of infections in the acute care setting. Am J Infect Control.

[88] Scheinemann K, Ethier MC, Dupuis LL, Richardson SE, Doyle J, Allen U, Sung L (2010). Utility of peripheral blood cultures in bacteremic pediatric cancer patients with a central line. Support Care Cancer.

[89] Handrup MM, Møller JK, Rutkjaer C, Schrøder H (2015). Importance of blood cultures from peripheral veins in pediatric patients with cancer and a central venous line. Pediatr Blood Cancer.

[90] Beutel K, Simon A (2005). Diagnostik und Therapie Katheter-assoziierter Infektionen in der pädiatrischen Onkologie. Klin Padiatr.

[91] Choi SW, Chang L, Hanauer DA, Shaffer-Hartman J, Teitelbaum D, Lewis I, Blackwood A, Akcasu N, Steel J, Christensen J, Niedner MF (2013). Rapid reduction of central line infections in hospitalized pediatric oncology patients through simple quality improvement methods. Pediatr Blood Cancer.

[92] Simon A, Bode U, Beutel K (2006). Diagnosis and treatment of catheter-related infections in paediatric oncology: an update. Clin Microbiol Infect.

[93] Maki DG, Weise CE, Sarafin HW (1977). A semiquantitative culture method for identifying intravenous-catheter-related infection. N Engl J Med.

[94] Gaur AH, Flynn PM, Shenep JL (2004). Optimum management of pediatric patients with fever and neutropenia. Indian J Pediatr.

[95] Horvath B, Norville R, Lee D, Hyde A, Gregurich M, Hockenberry M (2009). Reducing central venous catheter-related bloodstream infections in children with cancer. Oncol Nurs Forum.

[96] Soothill JS, Bravery K, Ho A, Macqueen S, Collins J, Lock P (2009). A fall in bloodstream infections followed a change to 2% chlorhexidine in 70% isopropanol for catheter connection antisepsis: a pediatric single center before/after study on a hemopoietic stem cell transplant ward. Am J Infect Control.

[97] Gaur AH, Flynn PM, Heine DJ, Giannini MA, Shenep JL, Hayden RT (2005). Diagnosis of catheter-related bloodstream infections among pediatric oncology patients lacking a peripheral culture, using differential time to detection. Pediatr Infect Dis J.

[98] Gaur A, Giannini MA, Flynn PM, Boudreaux JW, Mestemacher MA, Shenep JL, Hayden RT (2003). Optimizing blood culture practices in pediatric immunocompromised patients: evaluation of media types and blood culture volume. Pediatr Infect Dis J.

[99] Blot F, Schmidt E, Nitenberg G, Tancrède C, Leclercq B, Laplanche A, Andremont A (1998). Earlier positivity of central-venous- versus peripheral-blood cultures is highly predictive of catheter-related sepsis. J Clin Microbiol.

[100] Chen WT, Liu TM, Wu SH, Tan TD, Tseng HC, Shih CC (2009). Improving diagnosis of central venous catheter-related bloodstream infection by using differential time to positivity as a hospital-wide approach at a cancer hospital. J Infect.

[101] Wolf J, Shenep JL, Clifford V, Curtis N, Flynn PM (2013). Ethanol lock therapy in pediatric hematology and oncology. Pediatr Blood Cancer.

[102] Gaur AH, Miller MR, Gao C, Rosenberg C, Morrell GC, Coffin SE, Huskins WC (2013). Evaluating application of the National Healthcare Safety Network central line-associated bloodstream infection surveillance definition: a survey of pediatric intensive care and hematology/oncology units. Infect Control Hosp Epidemiol.

[103] Fraser JD, Aguayo P, Leys CM, Keckler SJ, Newland JG, Sharp SW, Murphy JP, Snyder CL, Sharp RJ, Andrews WS, Holcomb GW, Ostlie DJ, St Peter SD (2010). A complete course of intravenous antibiotics vs a combination of intravenous and oral antibiotics for perforated appendicitis in children: a prospective, randomized trial. J Pediatr Surg.

[104] Sexton DJ, Chen LF, Anderson DJ (2010). Current definitions of central line-associated bloodstream infection: is the emperor wearing clothes?. Infect Control Hosp Epidemiol.

[105] Fraser TG, Gordon SM (2011). CLABSI rates in immunocompromised patients: a valuable patient centered outcome?. Clin Infect Dis.

[106] Tomlinson D, Mermel LA, Ethier MC, Matlow A, Gillmeister B, Sung L (2011). Defining bloodstream infections related to central venous catheters in patients with cancer: a systematic review. Clin Infect Dis.

[107] Worth LJ, Slavin MA, Brown GV, Black J (2007). Catheter-related bloodstream infections in hematology: time for standardized surveillance?. Cancer.

[108] Freeman JT, Elinder-Camburn A, McClymont C, Anderson DJ, Bilkey M, Williamson DA, Berkahn L, Roberts SA (2013). Central line-associated bloodstream infections in adult hematology patients with febrile neutropenia: an evaluation of surveillance definitions using differential time to blood culture positivity. Infect Control Hosp Epidemiol.

[109] Rinke ML, Bundy DG, Chen AR, Milstone AM, Colantuoni E, Pehar M, Herpst C, Fratino L, Miller MR (2013). Central line maintenance bundles and CLABSIs in ambulatory oncology patients. Pediatrics.

[110] Rinke ML, Chen AR, Bundy DG, Colantuoni E, Fratino L, Drucis KM, Panton SY, Kokoszka M, Budd AP, Milstone AM, Miller MR (2012). Implementation of a central line maintenance care bundle in hospitalized pediatric oncology patients. Pediatrics.

[111] Rinke ML, Milstone AM, Chen AR, Mirski K, Bundy DG, Colantuoni E, Pehar M, Herpst C, Miller MR (2013). Ambulatory pediatric oncology CLABSIs: epidemiology and risk factors. Pediatr Blood Cancer.

[112] Gesetz zur Verhütung und Bekämpfung von Infektionskrankheiten beim Menschen (Infektionsschutzgesetz - IfSG). Infektionsschutzgesetz vom 20. Juli 2000 (BGBl. I S. 1045), das zuletzt durch Artikel 6a des Gesetzes vom 10. Dezember 2015 (BGBl. I S. 2229) geändert worden ist. 20.07.2000.

[113] Wiersma P, Schillie S, Keyserling H, Watson JR, De A, Banerjee SN, Drenzek CL, Arnold KE, Shivers C, Kendrick L, Ryan LG, Jensen B, Noble-Wang J, Srinivasan A (2010). Catheter-related polymicrobial bloodstream infections among pediatric bone marrow transplant outpatients--Atlanta, Georgia, 2007. Infect Control Hosp Epidemiol.

[114] Buchman AL, Opilla M, Kwasny M, Diamantidis TG, Okamoto R (2014). Risk factors for the development of catheter-related bloodstream infections in patients receiving home parenteral nutrition. JPEN J Parenter Enteral Nutr.

[115] Mohammed A, Grant FK, Zhao VM, Shane AL, Ziegler TR, Cole CR (2011). Characterization of posthospital bloodstream infections in children requiring home parenteral nutrition. JPEN J Parenter Enteral Nutr.

[116] Digiorgio MJ, Fatica C, Oden M, Bolwell B, Sekeres M, Kalaycio M, Akins P, Shane C, Bako J, Gordon SM, Fraser TG (2012). Development of a modified surveillance definition of central line-associated bloodstream infections for patients with hematologic malignancies. Infect Control Hosp Epidemiol.

[117] See I, Iwamoto M, Allen-Bridson K, Horan T, Magill SS, Thompson ND (2013). Mucosal barrier injury laboratory-confirmed bloodstream infection: results from a field test of a new National Healthcare Safety Network definition. Infect Control Hosp Epidemiol.

[118] Metzger KE, Rucker Y, Callaghan M, Churchill M, Jovanovic BD, Zembower TR, Bolon MK (2015). The burden of mucosal barrier injury laboratory-confirmed bloodstream infection among hematology, oncology, and stem cell transplant patients. Infect Control Hosp Epidemiol.

[119] Steinberg JP, Coffin SE (2013). Improving the central line-associated bloodstream infection surveillance definition: a work in progress. Infect Control Hosp Epidemiol.

[120] Haeusler GM, Phillips RS, Lehrnbecher T, Thursky KA, Sung L, Ammann RA (2015). Core outcomes and definitions for pediatric fever and neutropenia research: a consensus statement from an international panel. Pediatr Blood Cancer.

[121] Duarte RF, Greinix H, Rabin B, Mitchell SA, Basak G, Wolff D, Madrigal JA, Pavletic SZ, Lee SJ (2014). Uptake and use of recommendations for the diagnosis, severity scoring and management of chronic GVHD: an international survey of the EBMT-NCI Chronic GVHD Task Force. Bone Marrow Transplant.

[122] Carpenter PA, Kitko CL, Elad S, Flowers ME, Gea-Banacloche JC, Halter JP, Hoodin F, Johnston L, Lawitschka A, McDonald GB, Opipari AW, Savani BN, Schultz KR, Smith SR, Syrjala KL, Treister N, Vogelsang GB, Williams KM, Pavletic SZ, Martin PJ, Lee SJ, Couriel DR (2015). National Institutes of Health Consensus Development Project on Criteria for Clinical Trials in Chronic Graft-versus-Host Disease: V. The 2014 Ancillary Therapy and Supportive Care Working Group Report. Biol Blood Marrow Transplant.

[123] Worth LJ, Brett J, Bull AL, McBryde ES, Russo PL, Richards MJ (2009). Impact of revising the National Nosocomial Infection Surveillance System definition for catheter-related bloodstream infection in ICU: reproducibility of the National Healthcare Safety Network case definition in an Australian cohort of infection control professionals. Am J Infect Control.

[124] Beekmann SE, Diekema DJ, Huskins WC, Herwaldt L, Boyce JM, Sherertz RJ, Polgreen PM, Infectious Diseases Society of America Emerging Infections Network (2012). Diagnosing and reporting of central line-associated bloodstream infections. Infect Control Hosp Epidemiol.

[125] Sagana R, Hyzy RC (2013). Achieving zero central line-associated bloodstream infection rates in your intensive care unit. Crit Care Clin.

[126] Li S, Faustino EV, Golombek SG (2013). Reducing central line infections in pediatric and neonatal patients. Curr Infect Dis Rep.

[127] Waters HR, Korn R, Colantuoni E, Berenholtz SM, Goeschel CA, Needham DM, Pham JC, Lipitz-Snyderman A, Watson SR, Posa P, Pronovost PJ (2011). The business case for quality: economic analysis of the Michigan Keystone Patient Safety Program in ICUs. Am J Med Qual.

[128] Berenholtz SM, Lubomski LH, Weeks K, Goeschel CA, Marsteller JA, Pham JC, Sawyer MD, Thompson DA, Winters BD, Cosgrove SE, Yang T, Louis TA, Meyer Lucas B, George CT, Watson SR, Albert-Lesher MI, St Andre JR, Combes JR, Bohr D, Hines SC, Battles JB, Pronovost PJ, On the CUSP: Stop BSI program (2014). Eliminating central line-associated bloodstream infections: a national patient safety imperative. Infect Control Hosp Epidemiol.

[129] Gastmeier P, Sohr D, Geffers C, Nassauer A, Daschner F, Rüden H (2000). Are nosocomial infection rates in intensive care units useful benchmark parameters?. Infection.

[130] Dudeck MA, Weiner LM, Allen-Bridson K, Malpiedi PJ, Peterson KD, Pollock DA, Sievert DM, Edwards JR (2013). National Healthcare Safety Network (NHSN) report, data summary for 2012, Device-associated module. Am J Infect Control.

[131] Bundy DG, Gaur AH, Billett AL, He B, Colantuoni EA, Miller MR, Children’s Hospital Association Hematology/Oncology CLABSI Collaborative (2014). Preventing CLABSIs among pediatric hematology/oncology inpatients: national collaborative results. Pediatrics.

[132] Duffy EA, Rodgers CC, Shever LL, Hockenberry MJ (2015). Implementing a Daily Maintenance Care Bundle to Prevent Central Line-Associated Bloodstream Infections in Pediatric Oncology Patients. J Pediatr Oncol Nurs.

[133] Miller MR, Griswold M, Harris JM, Yenokyan G, Huskins WC, Moss M, Rice TB, Ridling D, Campbell D, Margolis P, Muething S, Brilli RJ (2010). Decreasing PICU catheter-associated bloodstream infections: NACHRI's quality transformation efforts. Pediatrics.

[134] Pronovost PJ, Goeschel CA, Colantuoni E, Watson S, Lubomski LH, Berenholtz SM, Thompson DA, Sinopoli DJ, Cosgrove S, Sexton JB, Marsteller JA, Hyzy RC, Welsh R, Posa P, Schumacher K, Needham D (2010). Sustaining reductions in catheter related bloodstream infections in Michigan intensive care units: observational study. BMJ.

[135] Reichardt C, Königer D, Bunte-Schönberger K, van der Linden P, Mönch N, Schwab F, Behnke M, Gastmeier P (2013). Three years of national hand hygiene campaign in Germany: what are the key conclusions for clinical practice?. J Hosp Infect.

[136] Scheithauer S, Lewalter K, Schröder J, Koch A, Häfner H, Krizanovic V, Nowicki K, Hilgers RD, Lemmen SW (2014). Reduction of central venous line-associated bloodstream infection rates by using a chlorhexidine-containing dressing. Infection.

[137] Weitz NA, Lauren CT, Weiser JA, LeBoeuf NR, Grossman ME, Biagas K, Garzon MC, Morel KD (2013). Chlorhexidine gluconate–impregnated central access catheter dressings as a cause of erosive contact dermatitis: a report of 7 cases. JAMA Dermatol.

[138] Zacharioudakis IM, Zervou FN, Arvanitis M, Ziakas PD, Mermel LA, Mylonakis E (2014). Antimicrobial lock solutions as a method to prevent central line-associated bloodstream infections: a meta-analysis of randomized controlled trials. Clin Infect Dis.

[139] Milstone AM, Elward A, Song X, Zerr DM, Orscheln R, Speck K, Obeng D, Reich NG, Coffin SE, Perl TM, Pediatric SCRUB Trial Study Group (2013). Daily chlorhexidine bathing to reduce bacteraemia in critically ill children: a multicentre, cluster-randomised, crossover trial. Lancet.

[140] Huang SS, Septimus E, Kleinman K, Moody J, Hickok J, Avery TR, Lankiewicz J, Gombosev A, Terpstra L, Hartford F, Hayden MK, Jernigan JA, Weinstein RA, Fraser VJ, Haffenreffer K, Cui E, Kaganov RE, Lolans K, Perlin JB, Platt R, CDC Prevention Epicenters Program, AHRQ DECIDE Network and Healthcare-Associated Infections Program (2013). Targeted versus universal decolonization to prevent ICU infection. N Engl J Med.

[141] Climo MW, Yokoe DS, Warren DK, Perl TM, Bolon M, Herwaldt LA, Weinstein RA, Sepkowitz KA, Jernigan JA, Sanogo K, Wong ES (2013). Effect of daily chlorhexidine bathing on hospital-acquired infection. N Engl J Med.

[142] Bass P, Karki S, Rhodes D, Gonelli S, Land G, Watson K, Spelman D, Harrington G, Kennon J, Cheng AC (2013). Impact of chlorhexidine-impregnated washcloths on reducing incidence of vancomycin-resistant enterococci colonization in hematology-oncology patients. Am J Infect Control.

[143] Kommission für Krankenhaushygiene und Infektionsprävention beim Robert Koch-Institut (2014). Empfehlungen zur Prävention und Kontrolle von Methicillin-resistenten Staphylococcus aureus-Stämmen (MRSA) in medizinischen und pflegerischen Einrichtungen. Bundesgesundheitsblatt Gesundheitsforschung Gesundheitsschutz.

[144] Pronovost P, Weast B, Rosenstein B, Sexton JB, Holzmueller CG, Paine L, Davies R, Rubin H (2005). Implementing and Validating a Comprehensive Unit-based Safety Program. J Patient Saf.

[145] Pronovost PJ, Berenholtz SM, Needham DM (2008). Translating evidence into practice: a model for large scale knowledge translation. BMJ.

[146] Saint S, Kowalski CP, Banaszak-Holl J, Forman J, Damschroder L, Krein SL (2010). The importance of leadership in preventing healthcare-associated infection: results of a multisite qualitative study. Infect Control Hosp Epidemiol.

[147] Wagner AK, Soumerai SB, Zhang F, Ross-Degnan D (2002). Segmented regression analysis of interrupted time series studies in medication use research. J Clin Pharm Ther.

[148] Furtwängler R, Laux C, Graf N, Simon A (2015). Impact of a modified Broviac maintenance care bundle on bloodstream infections in paediatric cancer patients. GMS Hyg Infect Control.

[149] Carraro F, Cicalese MP, Cesaro S, De Santis R, Zanazzo G, Tornesello A, Giordano P, Bergadano A, Giacchino M (2013). Guidelines for the use of long-term central venous catheter in children with hemato-oncological disorders. On behalf of supportive therapy working group of Italian Association of Pediatric Hematology and Oncology (AIEOP). Ann Hematol.

[150] Kommission für Krankenhaushygiene und Infektionsprävention beim Robert Koch-Institut (2013). Kommentar der Kommission für Krankenhaushygiene und Infektionsprävention (KRINKO): Aspekte der mikrobiologischen Diagnostik im Rahmen der Prävention von nosokomialen Infektionen. Epidemiol Bulletin des Robert Koch-Instituts.

[151] Kommission für Krankenhaushygiene und Infektionsprävention beim Robert Koch-Institut (2013). Praktische Umsetzung sowie krankenhaushygienische und infektionspräventive Konsequenzen des mikrobiellen Kolonisationsscreenings bei intensivmedizinisch behandelten Früh- und Neugeborenen. Ergänzende Empfehlung der KRINKO beim Robert Koch-Institut, Berlin, zur Implementierung der Empfehlungen zur Prävention nosokomialer Infektionen bei neonatologischen Intensivpflegepatienten mit einem Geburtsgewicht unter 1.500 g aus dem Jahr 2007 und 2012. Epidemiol Bulletin des Robert Koch-Instituts.

[152] Lehrnbecher T, Marshall D, Gao C, Chanock SJ (2002). A second look at anorectal infections in cancer patients in a large cancer institute: the success of early intervention with antibiotics and surgery. Infection.

[153] Vaiman M, Lazarovitch T, Heller L, Lotan G (2015). Ecthyma gangrenosum and ecthyma-like lesions: review article. Eur J Clin Microbiol Infect Dis.

[154] Vassallo M, Dunais B, Roger PM (2015). Antimicrobial lock therapy in central-line associated bloodstream infections: a systematic review. Infection.

[155] Saint S, Kowalski CP, Banaszak-Holl J, Forman J, Damschroder L, Krein SL (2009). How active resisters and organizational constipators affect health care-acquired infection prevention efforts. Jt Comm J Qual Patient Saf.

[156] De Bono S, Heling G, Borg MA (2014). Organizational culture and its implications for infection prevention and control in healthcare institutions. J Hosp Infect.

[157] Griffiths P, Renz A, Hughes J, Rafferty AM (2009). Impact of organisation and management factors on infection control in hospitals: a scoping review. J Hosp Infect.

[158] Kommission für Krankenhaushygiene und Infektionsprävention beim Robert Koch-Institut Berlin (2009). Personelle und organisatorische Voraussetzungen zur Prävention nosokomialer Infektionen. Empfehlung der Kommission für Krankenhaushygiene und Infektionsprävention. Bundesgesundheitsblatt Gesundheitsforschung Gesundheitsschutz.

[159] Robert Koch-Institut Berlin (Bundesgesundheitsblatt). Bekanntmachung des Robert Koch-Instituts: Festlegung der Daten zu Art und Umfang des Antibiotika-Verbrauchs in Krankenhäusern nach § 23 Abs. 4 Satz 2 IfSG. Vom RKI gemäß § 4 Abs. 2 Nr. 2b zu erstellende Liste über die Daten zu Art und Umfang des Antibiotika-Verbrauchs.

[160] Hugonnet S, Harbarth S, Sax H, Duncan RA, Pittet D (2004). Nursing resources: a major determinant of nosocomial infection?. Curr Opin Infect Dis.

[161] Resar RK (2006). Making noncatastrophic health care processes reliable: Learning to walk before running in creating high-reliability organizations. Health Serv Res.

[162] Lynn J, Baily MA, Bottrell M, Jennings B, Levine RJ, Davidoff F, Casarett D, Corrigan J, Fox E, Wynia MK, Agich GJ, O'Kane M, Speroff T, Schyve P, Batalden P, Tunis S, Berlinger N, Cronenwett L, Fitzmaurice JM, Dubler NN, James B (2007). The ethics of using quality improvement methods in health care. Ann Intern Med.

[163] Zingg W, Cartier V, Inan C, Touveneau S, Theriault M, Gayet-Ageron A, Clergue F, Pittet D, Walder B (2014). Hospital-wide multidisciplinary, multimodal intervention programme to reduce central venous catheter-associated bloodstream infection. PLoS ONE.

[164] Zingg W, Imhof A, Maggiorini M, Stocker R, Keller E, Ruef C (2009). Impact of a prevention strategy targeting hand hygiene and catheter care on the incidence of catheter-related bloodstream infections. Crit Care Med.

[165] Hansen S, Schwab F, Schneider S, Sohr D, Gastmeier P, Geffers C (2014). Time-series analysis to observe the impact of a centrally organized educational intervention on the prevention of central-line-associated bloodstream infections in 32 German intensive care units. J Hosp Infect.

[166] Urrea M, Rives S, Cruz O, Navarro A, García JJ, Estella J (2004). Nosocomial infections among pediatric hematology/oncology patients: results of a prospective incidence study. Am J Infect Control.

[167] Carlisle PS, Gucalp R, Wiernik PH (1993). Nosocomial infections in neutropenic cancer patients. Infect Control Hosp Epidemiol.

[168] Barrell C, Covington L, Bhatia M, Robison J, Patel S, Jacobson JS, Buet A, Graham PL, Saiman L (2012). Preventive strategies for central line-associated bloodstream infections in pediatric hematopoietic stem cell transplant recipients. Am J Infect Control.

[169] Berrueco R, Rives S, Català A, Toll T, Gene A, Ruiz A, Badosa R, Claramonte MA, Estella J, Urrea M (2013). Prospective surveillance study of blood stream infections associated with central venous access devices (port-type) in children with acute leukemia: an intervention program. J Pediatr Hematol Oncol.

[170] Winters BD, Gurses AP, Lehmann H, Sexton JB, Rampersad CJ, Pronovost PJ (2009). Clinical review: checklists - translating evidence into practice. Crit Care.

[171] Bosk CL, Dixon-Woods M, Goeschel CA, Pronovost PJ (2009). Reality check for checklists. Lancet.

